# Inhibition of Pediatric Glioblastoma Tumor Growth by the Anti-Cancer Agent OKN-007 in Orthotopic Mouse Xenografts

**DOI:** 10.1371/journal.pone.0134276

**Published:** 2015-08-06

**Authors:** Patricia Coutinho de Souza, Samantha Mallory, Nataliya Smith, Debra Saunders, Xiao-Nan Li, Rene Y. McNall-Knapp, Kar-Ming Fung, Rheal A. Towner

**Affiliations:** 1 Advanced Magnetic Resonance Center, Oklahoma Medical Research Foundation, Oklahoma City, OK, United States of America; 2 Department of Veterinary Pathobiology, College of Veterinary Medicine, Oklahoma State University, Stillwater, OK, United States of America; 3 University of Oklahoma Children's Hospital, Oklahoma City, OK, United States of America; 4 Laboratory of Molecular Neuro-Oncology, Texas Children's Cancer Center, Texas Children's Hospital, Houston, TX, United States of America; 5 Peggy and Charles Stephenson Cancer Center, Oklahoma City, OK, United States of America; 6 Department of Pathology, University of Oklahoma Health Sciences Center, Oklahoma City, OK, United States of America; 7 Department of Pathology, Oklahoma City Veterans Affairs Medical Center, Oklahoma City, OK, United States of America; University Hospital of Navarra, SPAIN

## Abstract

Pediatric glioblastomas (pGBM), although rare, are one of the leading causes of cancer-related deaths in children, with tumors essentially refractory to existing treatments. Here, we describe the use of conventional and advanced *in vivo* magnetic resonance imaging (MRI) techniques to assess a novel orthotopic xenograft pGBM mouse (IC-3752GBM patient-derived culture) model, and to monitor the effects of the anti-cancer agent OKN-007 as an inhibitor of pGBM tumor growth. Immunohistochemistry support data is also presented for cell proliferation and tumor growth signaling. OKN-007 was found to significantly decrease tumor volumes (*p*<0.05) and increase animal survival (*p*<0.05) in all OKN-007-treated mice compared to untreated animals. In a responsive cohort of treated animals, OKN-007 was able to significantly decrease tumor volumes (p<0.0001), increase survival (p<0.001), and increase diffusion (*p*<0.01) and perfusion rates (*p*<0.05). OKN-007 also significantly reduced lipid tumor metabolism in responsive animals [(Lip1.3 and Lip0.9)-to-creatine ratio (*p*<0.05)], as well as significantly decrease tumor cell proliferation (*p*<0.05) and microvessel density (*p*<0.05). Furthermore, in relationship to the PDGFRα pathway, OKN-007 was able to significantly decrease SULF2 (*p*<0.05) and PDGFR-*α* (platelet-derived growth factor receptor*-α*) (*p*<0.05) immunoexpression, and significantly increase decorin expression (*p*<0.05) in responsive mice. This study indicates that OKN-007 may be an effective anti-cancer agent for some patients with pGBMs by inhibiting cell proliferation and angiogenesis, possibly via the PDGFR*α* pathway, and could be considered as an additional therapy for pediatric brain tumor patients.

## Introduction

High grade gliomas (HGG) represent one of the most common central nervous system (CNS) tumors among adults. This contrasts significantly to the pediatric population where HGGs only comprise approximately 8–12% of all primary CNS tumors [1]. These tumors are classified by the World Health Organization (WHO) as either grade III or IV meaning that they are highly malignant tumors with characteristic features such as hypercellularity, nuclear atypia, and high mitotic activity with or without microvascular proliferation and necrosis [2]. The most common pediatric HGGs include anaplastic astrocytoma (WHO grade III) and glioblastoma (GBM; WHO grade IV). Pediatric glioblastoma (pGBM) is one of the leading causes of cancer-related deaths in children [3] with a median overall survival for patients of 11 months [4]. Despite improvements in neurosurgery, radiotherapy, and chemotherapy, the outcome for children with pediatric HGG remains poor [5]. No effective chemotherapy regimens have been identified for any pediatric HGG yet [5]. Furthermore, novel molecularly targeted agents have demonstrated little efficacy in early phase clinical trials [6–8], highlighting the need for new treatment approaches.

Similarly, the improvement of *in vitro* and *in vivo* models for pediatric HGGs is also needed. Tumor cell lines and animal models derived from CNS tumors are essential tools to study the biology of the disease, to comprehend the mechanisms of resistance to therapy, and to carry out preclinical therapeutic testing. There is abundant information on animal models of adult HGG [9–11], including chemically induced, genetically engineered mice, and xenograft models [9]. However, to date only a few pediatric GBM models have been established and/or characterized [12–14].

We have shown that the anti-cancer agent OKN-007 (OKlahoma Nitrone-007; disodium 4-[(tert-butyl-imino) methyl] benzene-1,3-disulfonate N-oxide or disufenton) is very effective against adult gliomas in preclinical models [11,15,16], and it is currently undergoing clinical trial assessment as a new investigational drug for recurrent adult glioblastomas. OKN-007 is a small molecule that can traverse the blood-brain barrier and has anti-inflammatory, antioxidant, and proapoptotic properties [17,18]. In an orthotopic rat F98 glioma model and a human U87 xenograft model in athymic rats, OKN-007 significantly increased the survival of treated versus untreated rats [16]. Tumor volumes in both of these models were significantly decreased in treated compared to untreated animals based on magnetic resonance imaging (MRI) assessment [16]. Immunohistochemistry (IHC) assessment of commonly studied tumor markers for cell proliferation or differentiation, hypoxia, angiogenesis, and apoptosis indicated that OKN-007 was able to significantly decrease cell proliferation (glucose transporter 1 (Glut-1) and the cell proliferation marker, MIB-1) but not cell differentiation (carbonate anhydrase IX), decrease angiogenesis (microvessel density (MVD; measured as levels of the endothelial marker, CD-31), but not the vascular endothelial growth factor (VEGF)), decrease hypoxia (hypoxia inducible factor 1 α (HIF-1α)), and increase apoptosis (cleaved caspase 3) compared with untreated controls [16]. OKN-007-induced decreases in Glut-1 and HIF-1α levels seemed to be similar in both F98 and U87 glioma models, whereas increased apoptosis seemed to be more elevated in the F98 gliomas compared to the U87 tumors [16]. We concluded that OKN-007 has the ability to cause glioma regression in aggressive rodent tumor models (F98 and U87), as well as in moderate gliomas (C6) [11,15,16]. Support for the anti-cancer effect of OKN-007 via the TGFβ1 pathway was recently reported by Zheng *et al*. (2013), where they found that OKN-007 mediates its antitumor effect in HCC cells (Huh7) via the suppression of TGFβ1/SMAD2 and Hedgehog/GLI1 signaling by inhibiting sulfatase 2 (SULF2) enzymatic activity [19].

PDGFR*α* is the most frequent target of focal amplification in pediatric HGGs [20–23]. Somatic mutations of PDGFR*α* have been also recently reported in pediatric HGGs [22–24]. It is also known that the SULF2 gene expression is correlated with PDGFR*α* expression [25]. Similarly to SULF2, decorin is also associated to the PDGFRα pathway [26]. Since it was previously shown that OKN-007 could affect SULF2 activity, we thought that perhaps this agent could be effective against pediatric HGGs.

MRI has been used in preclinical [12] and clinical [27–32] studies to diagnose and predict therapy outcomes for pediatric GBMs. The techniques most commonly used include *in vivo* ^1^H magnetic resonance spectroscopy (^1^H-MRS) [33–35], diffusion-weighted imaging (DWI) [36], and arterial spin labeling perfusion (ASL) [37]. ^1^H-MRS has been used in research and clinical settings to measure metabolite levels in the brain, and is well suited to perform the diagnosis, characterization, and assessment of tumor response to treatment [38]. DWI can qualitatively and quantitatively assess molecular motion on the basis of organization of tissue microstructures by characterizing water mobility [39], and can provide a more complete evaluation of cellular and metabolic information about human brain cancer [40]. ASL allows the assessment of measuring tumor blood flow [41], which assists in the characterization of tumor grade [37] and response to cancer therapy [42].

Here we describe the MRI characterization of a novel orthotopic xenograft pediatric HGG mouse (IC-3752GBM patient-derived primary culture) model and the effects of OKN-007 to inhibit *in vivo* pediatric glioblastoma IC-3752GBM tumor growth using conventional (morphological imaging) and advanced MRI techniques (^1^H-MRS, DWI, and ASL). Immunohistochemical staining was also used to evaluate the levels of tumor-related proteins and to assess tumor cell proliferation. To the best of our knowledge, the current study is the first report of MRI characterization of an orthotopic xenograft murine model of pGBM, and the assessment of the anti-cancer agent, OKN-007, in a pGBM model.

## Materials and Methods

### Ethics Statement

All experiments of the current study were performed following a human protocol approved by the Institutional Review Board (IRB) at Baylor College of Medicine as well as an animal protocol approved by the Institutional Animal Care and Use Committee (IACUC) of Baylor College of Medicine and the IACUC of the Oklahoma Medical Research Foundation. For human tissue samples, signed informed consent was obtained from all patients or their legal guardians prior to sample acquisition.

### Brain tumor specimen

A fresh tumor specimen (3752GBM), from a 5-year-old female who underwent craniotomy at the Texas Children's Hospital, was obtained in a cryostat laboratory with signed consent following an Institutional Review Board-approved protocol (H-4844). Final pathological diagnosis was consistent with pediatric glioblastoma (pGBM) [43]. The tumor sample was directly implanted into right cerebral cortex of immunedeficient mice (Baylor College of Medicine IACUC # AN-4548). Xenograft tumor cells harvested from donor mice were passaged in nude mice via six subsequent implantations as we described previously [43,44].

### Intracranial mouse brain tumor model

The passaged pediatric glioblastoma culture IC-3752GBM was implanted intracerebrally in athymic nude mice (n = 24) (OMRF IACUC protocols # 13–51 and # 13–53). The heads of anesthetized mice were immobilized (stereotaxic unit; Kopf Instruments, Tujunga, CA), and with aseptic techniques, a 1 mm burr hole was drilled in the skull 1 mm anterior and 2 mm lateral to the bregma on the left or right side. A 20 μL gas-tight Hamilton syringe was used to inject 5–10 × 10^6^ IC-3752GBM cells/mL suspended in 4 μL in cell culture media with 1.0% agarose into the right or left frontal lobe at a depth of 1.5 mm relative to the dural surface in a stereotaxic unit. Analgesia (Buprenex, i.p. injection) was used following the cell implantation procedure for pain relief during recovery. The cell lines were maintained and expanded immediately prior to inoculation. Following injection, the skin was closed with surgical sutures. The animals were divided into two groups: OKN-007–treated (n = 12) and untreated (n = 12) groups. Both groups were stratified to ensure that tumor sizes were similar before treatment started in the treated group. The IC-3752GBM primary culture was not passaged more than six times in mice.

### OKN-007 treatment

OKN-007 (Ryss Laboratories, Union City, CA) was administered to the mice in their drinking water at a concentration of 150 mg/kg/day (0.20% w/v for a 20g mouse). The treatment started when the tumor volumes were between 10 and 15 mm^3^, and was administered continuously until the end of the study. Mice receiving normal drinking water were used as untreated controls. The amount of OKN-007 consumed by each mouse, which were housed in separate cages, was determined by weighing water bottles each day. No significant deviation was observed in the volume of liquid uptake of compound in these mice. The average intake of OKN-007 was approximately 130–150 mg/kg/day for all mice. For the entire study, the therapeutic response to OKN-007 was evaluated as either responsive (OKN-007-R) or non-responsive (OKN-007-NR). Mortality was recorded daily during the study period to calculate the percentage survival of all animals. All animals were humanely euthanized (CO_2_ asphyxiation) when they met tumor burden criteria (tumors ≥ 150 mm^3^) and/or showed signs of illness, weight loss, poor body condition, porphyria, hypoactivity, restlessness, aggressiveness, ataxia, shallow, rapid and/or labored breathing, cachexia, failure to respond to stimuli, lack of inquisitiveness, vocalization, seizures, hunched posture and ruffled fur). Animals were monitored twice daily.

### Magnetic resonance techniques

#### Morphological imaging

Nude mice were anesthetized and positioned in a stereotaxic cradle. A 30-cm horizontal bore Bruker Biospin magnet operating at 7 Tesla (T; Bruker BioSpin GmbH, Karlsruhe, Germany), was used with a S116 gradient set to perform all MRI experiments. An EPI (echo planar imaging) transceiver ^1^H 50W coil with a 38.0 mm inner diameter was used for signal transmission and detection. Multiple-slice, multiple echo (MSME) imaging (FOV = 2.50 x 2.50 cm^2^, TR = 2000 ms, TE = 17.5 or 58.2 ms, matrix = 192, averages = 2, slices = 16, slice thickness = 1 mm) was used to calculate tumor volumes and to inspect tumor morphology. Multi slice spin echo T_1_-weighted images (TR = 1000.0 ms, TE = 14 ms, FOV = 2.50 × 2.50 cm^2^, averages = 2, slices = 16, matrix size = 256 × 256) were also performed and acquired before and 15 min after intravenous contrast agent injection (Gd-DTPA, Magnevist, Bayer Inc., Wayne, NY, USA; 0.4mmol/kg).

#### ^1^H-MR Spectroscopy

^1^H-MRS was acquired using a PRESS (Point REsolved SpectroScopy) sequence with a TE of 24.0 ms, a TR of 2500.0 ms, 512 averages, and a spectral width of 4006 Hz. A non-suppressed MR spectrum was acquired beforehand by applying eddy-current correction to maximize signal intensity and decrease the peak linewidths. Water was suppressed with a VAPOR (variable power radio frequency pulses and optimized relaxation delays) suppression scheme. In all cases, the peak width (full width at half maximum) of the water peak was less than 30 Hz following localized shimming, which was conducted by using first and second order adjustments with Fastmap. A cubic voxel of 2.0 x 2.0 x 2.0 mm^3^ was positioned in either the tumor or the contralateral normal brain tissue, while maximizing the amount of tumor tissue present in the voxel at all times.

To analyze the MRS data, an in-house Mathematica program was used (version 6.0, Wolfram Research, Champaign, IL, USA). The spectra were scaled in ppm by calibrating against the water peak (4.78 ppm). The major brain metabolic peaks were identified as: N-acetylaspartate (NAA) at 2.02 ppm, choline (Cho) at 3.22 ppm, creatine (Cre) at 3.02 ppm, and mobile lipids at 1.3 ppm (Lip1.3) for the methylene group (-CH_2_-) and 0.9 ppm (Lip0.9) for the methyl group (-CH_3_) in the tumor tissue. The peak area measurements of the metabolites were used to calculate the following ratios: tumor NAA to tumor Cho (NAA^t^/Cho^t^), tumor Cho to contralateral (normal tissue) Cre (Cho^t^/Cre^n^), tumor Cho to tumor NAA (Cho^t^/NAA^t^), tumor Lip0.9 to contralateral Cre (Lip0.9^t^/Cre^n^), and tumor Lip1.3 to contralateral Cre (Lip1.3^t^/Cre^n^).

#### Diffusion-Weighted Imaging

A coronal axial multi-slice DWI sequence covering the entire tumor was performed using an echo planar imaging—based pulse sequence with the following parameters: TR = 4000 ms, TE = 51.1 ms, matrix size = 128 × 128, slice thickness = 1mm, diffusion gradient duration = 4 ms, diffusion gradient separation = 14 ms. Five images were obtained with different gradient scalings, resulting in b-values of 100, 200, 500, 700, and 850 s/mm^−2^. ADC maps were generated for all the slices in which the tumor was observed. The apparent diffusion coefficient (ADC) values were obtained by drawing a single freehand ROI along the border of the tumor normalized to the contralateral normal brain.

#### Arterial spin-labeling perfusion

Perfusion maps were obtained on a single axial slice of the brain located on the point of the rostro—caudal axis where the tumor had the largest cross-section. The imaging geometry was a 25.6 × 25.6 mm^2^ field-of-view (FOV) of 2 mm in thickness, with a single shot echo-planar encoding over a 64 × 64 matrix. An echo time (TE) of 13.5 ms, a repetition time (TR) of 18 s, and an inversion time (TIR) of 26.0 ms were used, and images were not submitted to time averaging. To obtain perfusion contrast, the flow alternating inversion recovery scheme was used. Briefly, inversion recovery images were acquired using a slice-selective inversion of the same geometry as the imaging slice or a nonselective inversion slice concentric with the imaging slice with a slice package margin of 5.0 mm. For each type of inversion, 22 images were acquired with inversion times evenly spaced from 26.0 ms to 8,426.0 ms (with an increment of 400 ms between each TIR). CBV (cerebral blood flow) values were obtained by drawing a single freehand ROI along the border of the tumor normalized to the contralateral normal brain to obtain normalized relative CBF (rCBF) values. Negative ASL CBF values were assumed to be zero [45].

### Gross and microscopic histology

Necropsies were performed on all mice. The extent of tumor at necropsy was evaluated by gross and microscopic examination of the brain for each animal from the untreated and OKN-007–treated groups. Brain tissues were fixed in 10% phosphate buffered formalin, embedded in paraffin, serially sectioned at 4 μm, and were stained with hematoxylin eosin (H&E) for histological examination.

### Immunohistochemistry

Immunohistochemical staining was performed using an automated immunostainer (Leica, Bond-III, Leica, Buffalo Grove, IL) with the following primary polyclonal antibodies: platelet-derived growth factor receptor alpha (PDGFR*α*) (1:1000 dilution, rabbit polyclonal, clone ab61219, ABCAM), decorin (1:500 dilution, rabbit polyclonal, clone LS-B 8177 / 45156), LSBIO LifeSpan BioSciences), heparan sulfate sulfatase SULF2 (1:100 dilution, rabbit polyclonal, clone AV49338, Sigma Aldrich), and CD31 (1:25 dilution, rabbit polyclonal, clone ab28364, ABCAM, Ki-67 (1:100 dilution, rabbit polyclonal, clone PA5-19462, Thermo Fisher Scientific, IL). All antibodies were initially optimized on control tissues, and positive and negative controls were processed in parallel to the tissue sections.

### IHC Scoring

All IHCs were analyzed using the Aperio ScanScope Image Analysis System (Aperio, Vista, CA). PDGFR*α*, SULF2, and decorin IHCs were analyzed using a Positive Pixel Count algorithm with the Aperio ImageScope viewer. Only areas containing tumor tissue were analyzed for IHC expression. Areas without tumor tissue and areas with necrosis or significant artifacts (e.g. tissue folding) were deselected and excluded from analysis. The number of positive pixels from regions-of-interest (ROIs) with high expression were divided by the total number of pixels (negative and positive) in the analyzed area, and multiplied by 100, to derive the percentage of positive pixels.

Microvessel density (MVD, number of vessels per mm^2^) and Mean Vessel Area (MVA, Vascular area/2 + lumen area), using CD31 as a marker, were determined using an Aperio microvessel analysis algorithm. Three representative non-overlapping regions of interest (ROIs) for tumor samples were randomly selected, and the MVD and MVA were quantified for each animal from both untreated and OKN-007–treated groups.

An Aperio ScanScope Image Analysis System was also used to determine the KI-67 labeling index (Ki-67 LI). Three ROIs with the highest number of labeled nuclei were identified in each case. Ki-67 LI was determined by counting 1000 cells and expressing this as the number of labeled cells per 1000 cells [46] for each animal from both untreated and OKN-007–treated groups. Labeled cells adjacent to or within necrotic areas were also excluded while counting the Ki-67 LI. The means and standard deviations of Ki-67 LI were determined for each group.

### Statistical Analysis

Statistical analyses were performed using Graph Pad Prism 6 (GraphPad Prism 6 Software, San Diego, CA, USA). All *p* values <0.05 were considered statistically significant. Tumor volumes, metabolite peak length ratios [(NAA^t^/Cho^t^), (Cho^t^/Cre^n^), (Cho^t^/NAA^t^), (Lip0.9^t^/Cre^n^), and (Lip1.3^t^/Cre^n^)], normalized ADC, rCBF, Ki-67 LI, and immunoexpression of PDGFR*α*, SULF2, and decorin were reported as means ± standard deviations. For statistical analysis, Student t-tests (independent-samples, two-tailed t-test) were used to assess the differences between means of the normal, untreated, and OKN-007 treated pediatric IC-3752GBM glioma mice. Kaplan-Meier survival curves and a Log-rank (mantel-cox) test were used to compare the survival times among the untreated and OKN-007 treated groups.

## Results

Macroscopic, histological, immunohistochemical and MRI characteristics of the novel orthotopic xenograft pediatric glioblastoma (pGBM) model IC-3752GBM and the effects of OKN-007 to inhibit IC-3752GBM tumor culture growth were described in this report.

**Fig 1:**
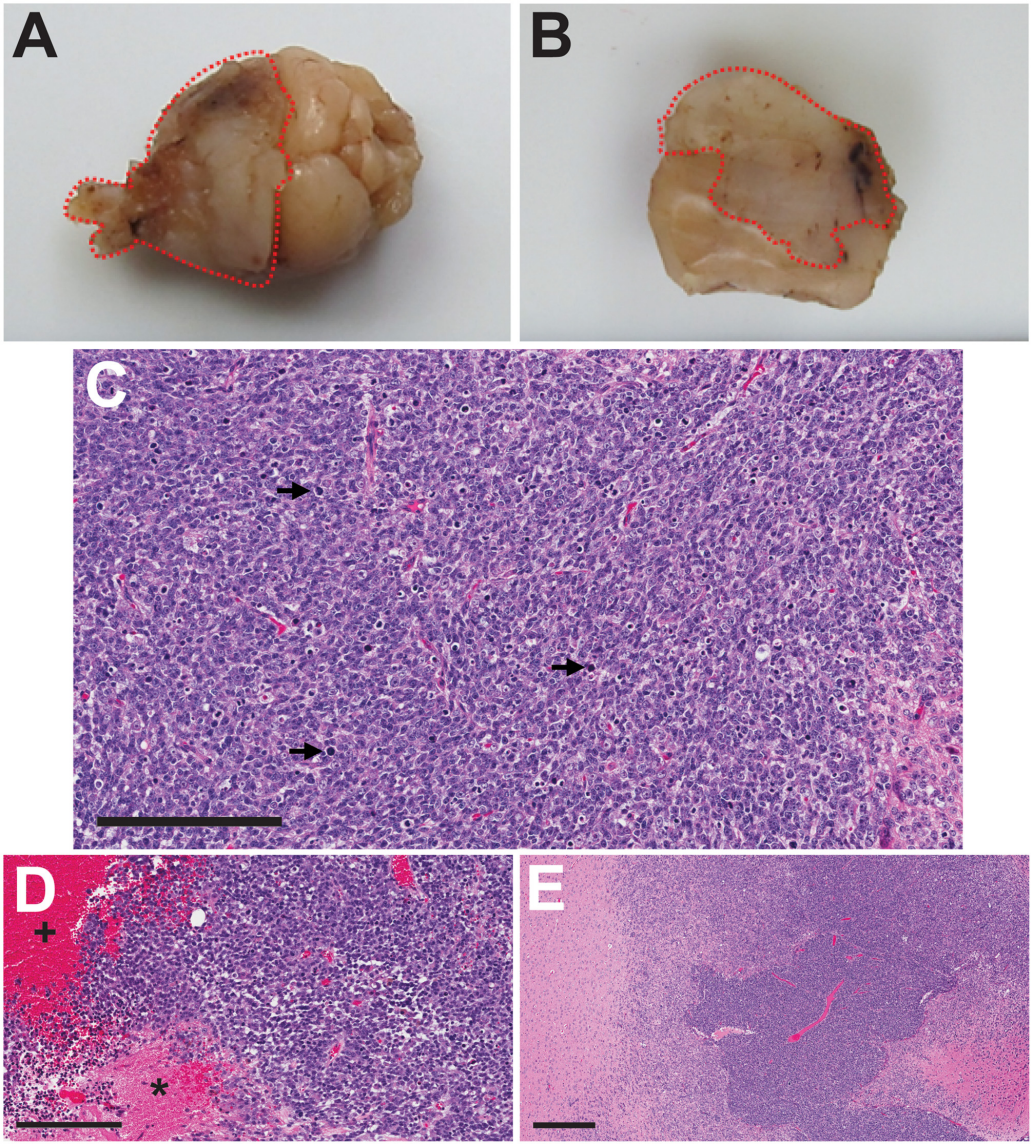
Gross and microscopic pathology of the IC3752 pediatric glioma tumor. (A-B) Formalin fixed IC3752 pGBM tissue, characterized as a yellowish gray and poorly demarcated mass, with multifocal areas of necrosis and hemorrhage. (C-E) Histological examination revealed poorly demarcated, unencapsulated, and invasive neoplasms (E) characterized by marked cell atypia, a high mitotic index (black arrows) (C), and multifocal areas of necrosis (asterisks) and hemorrhage (plus sign) (D). T = Tumor, B = normal brain. Scale bar = 200 μm (C-D). Scale bar = 400 μm (E).

Gross and microscopic characteristics of the IC-3752GBM pGBM model are presented in Fig 1A–1E. Macroscopically the tumors were characterized as a yellowish gray, poorly demarcated mass, with multifocal areas of necrosis and hemorrhage. Histological examination revealed poorly demarcated, unencapsulated infiltrating neoplasm with marked cell atypia, high mitotic index, and multifocal areas of necrosis and hemorrhage.

**Fig 2:**
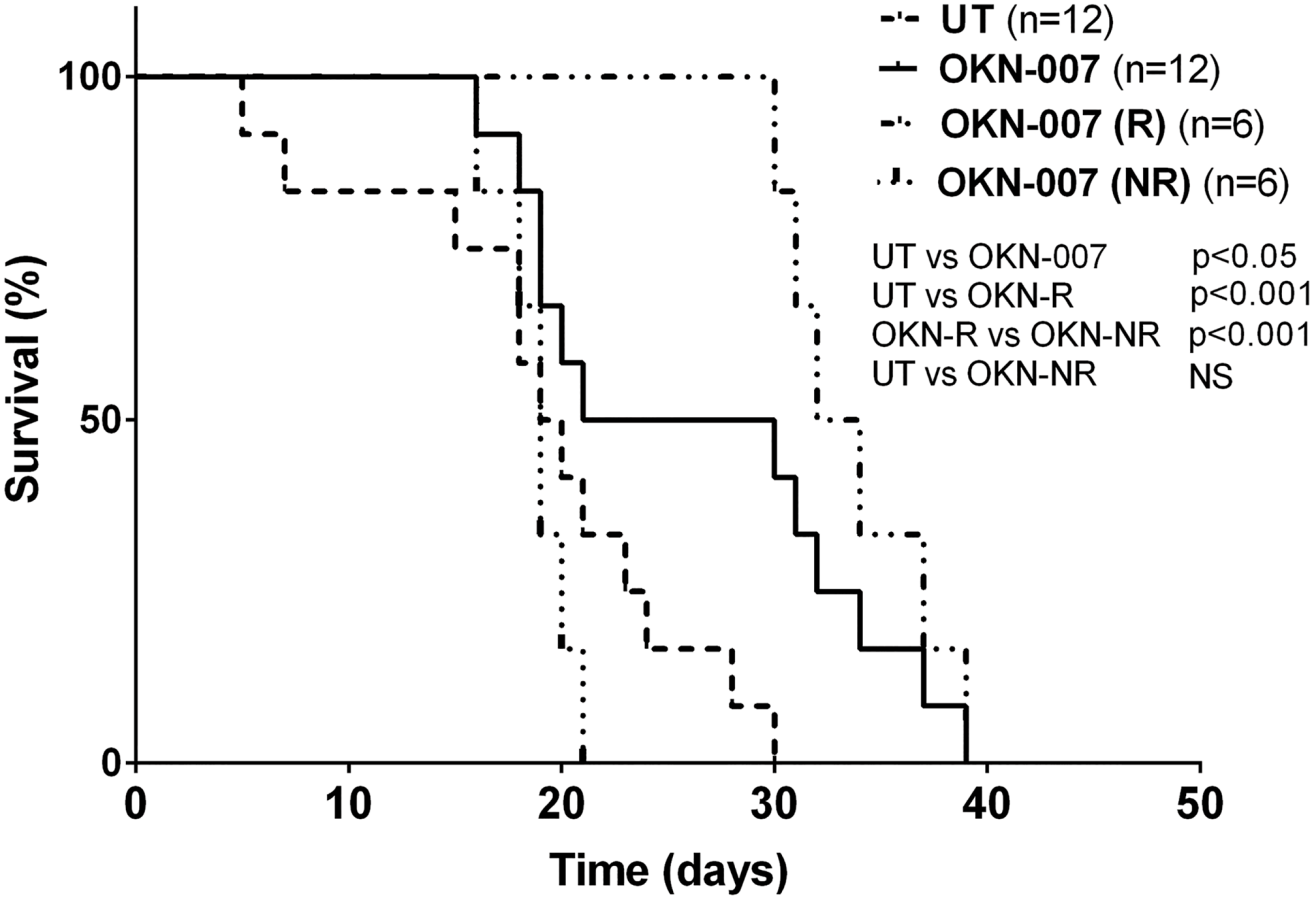
Effect of OKN-007 on percent survival for IC3752 pGBM tumor bearing mice. There was a significant increase in percent survival for all OKN-007-treated mice (n = 12) (*p* < 0.05) compared to untreated tumor-bearing mice (n = 12). Based on the different response cohorts, the OKN-007-treatment group was further divided into OKN-007-R (responsive) and OKN-007-NR (non-responsive) cohorts. OKN-007-R mice (n = 6) demonstrated significantly longer survival times (*p* < 0.001) when compared with UT animals (n = 12). Conversely, OKN-007-NR mice (n = 6) showed no statistical difference (*p* > 0.05) when compared to the UT group.

Animal survival was assessed by comparing OKN-007–treated animals with untreated animals (Fig 2). Overall, OKN-007 was found to significantly increase animal survival (p<0.0223) (median survival of 25.5 days) compared to untreated tumor-bearing mice (median survival of 19.5 days). The total OKN-007-treated survival data seemed to depict two treatment response cohorts. For instance, it was found that six animals responded to the OKN-007 treatment and demonstrated significantly longer survival times (*p* = 0.0002) (median survival of 33 days) when compared with untreated animals (n = 12). Conversely, six other pGBM mice did not seem to respond to the OKN-007 therapy, and showed no statistical difference (*p* = 0.3392) when compared to the untreated group. The non-responsive OKN-007-treated mice seemed to have a median survival (19 days) that was similar to the median survival for the untreated tumor-bearing mice.

**Fig 3:**
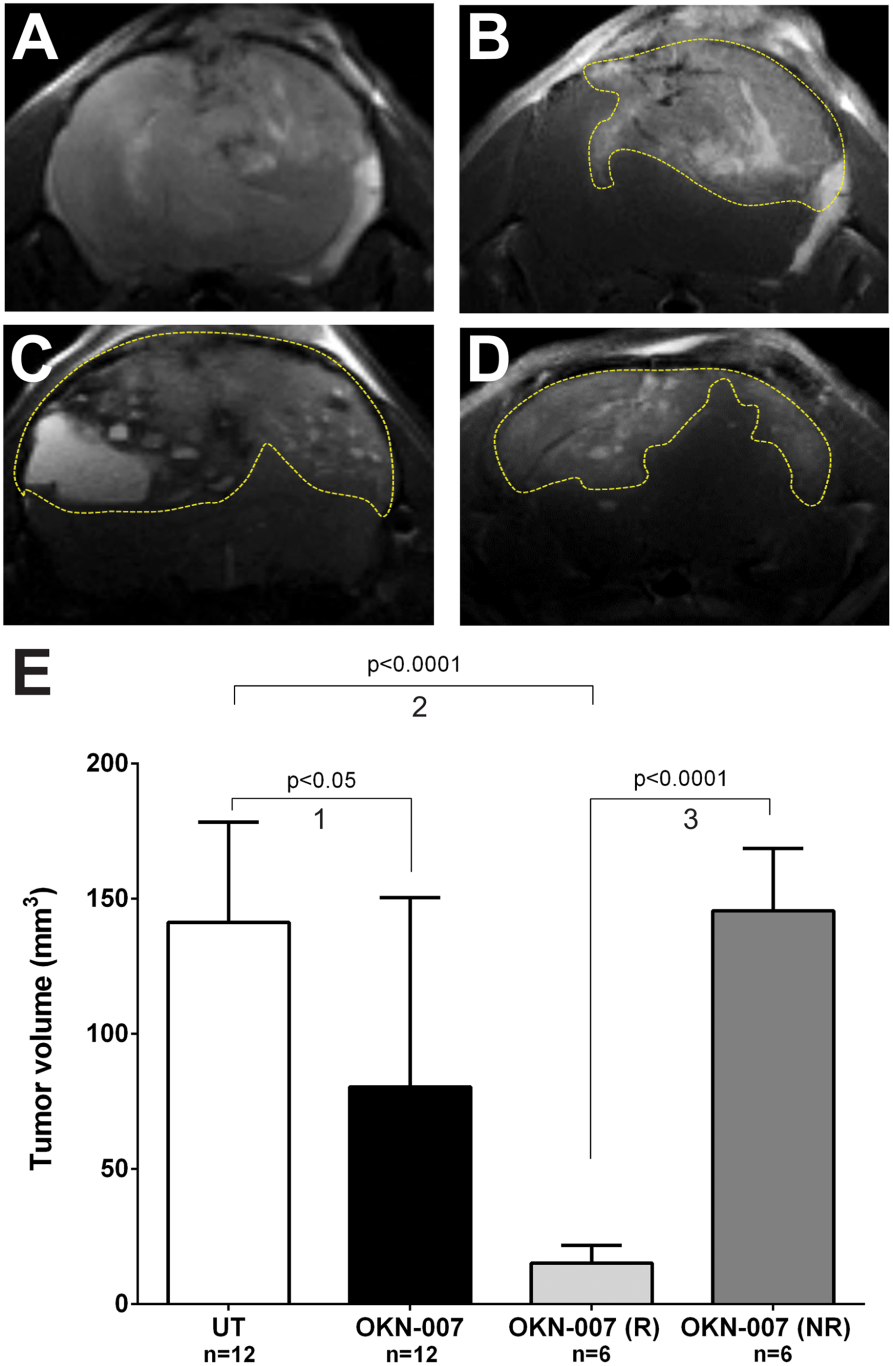
Effect of OKN-007 on tumor volumes for IC3752 pGBM tumor bearing mice. Representative T1-weighted morphological images before (A) and after contrast agent (Gd-DTPA) injection (B) in pGBM-bearing mice are shown. (A) Before contrast administration the tumor appeared as a mildly hyperintense mass and it was difficult to demarcate it. (B) Fifteen minutes after the contrast injection the IC3752 pGBM demonstrates enhancement, delineating the tumor (yellow dashed line). Representative T2-weighted MR images of UT (C) and OKN-007 (D) treated IC 3752 pGBM-bearing mouse brains. (E) The quantitative histogram for all mice shows that all OKN-007-treated mice (n = 12) had a significantly decreased tumor volume (p < 0.05) compared to UT mice (n = 12). Six animals responded to the OKN-007 treatment [OKN-007 (R)] and demonstrated significantly smaller tumors (*p* < 0.0001) when compared with UT animals (n = 12). However, six other pGBM mice did not seem to respond [OKN-007 (NR)] to the OKN-007 therapy, and there was no statistical difference between the UT and OKN-007 (NR) group. Values are represented as means ± SD. Group comparisons: (1) UT vs. OKN-007 (total); (2) UT vs. OKN-007 (R); and (3) OKN-007 (R) vs OKN-007 (NR).

To calculate the whole tumor volume in some animals, MSME T_1_-weighted images were performed and acquired before (Fig 3A) and 15 min after (Fig 3B) intravenous contrast agent injection (Gd-DTPA, Magnevist, Bayer Inc., Wayne, NY, USA). Fig 3E shows the quantitative mean IC-3752GBM tumor volumes calculated from MR images. The total OKN-007-treatment group was found to significantly decrease tumor volumes (p = 0.0289) compared to untreated mice (Fig 3E). Based on the survival data which indicated responsive and non-responsive OKN-007 treatment cohorts, the tumor volume was also separated in terms of treatment response. Six animals were found to respond to OKN-007 treatment [OKN-007-(R)] and demonstrated significantly smaller tumors (*p* <0.001) (Fig 3D) when compared with untreated animals (n = 12) (Fig 3C). It was also found that six pGBM mice found not to respond [OKN-007-(NR)] to the OKN-007 therapy (based from animal survival data), showed statistical difference (*p* = 0.7857) when compared to the untreated group.

**Fig 4:**
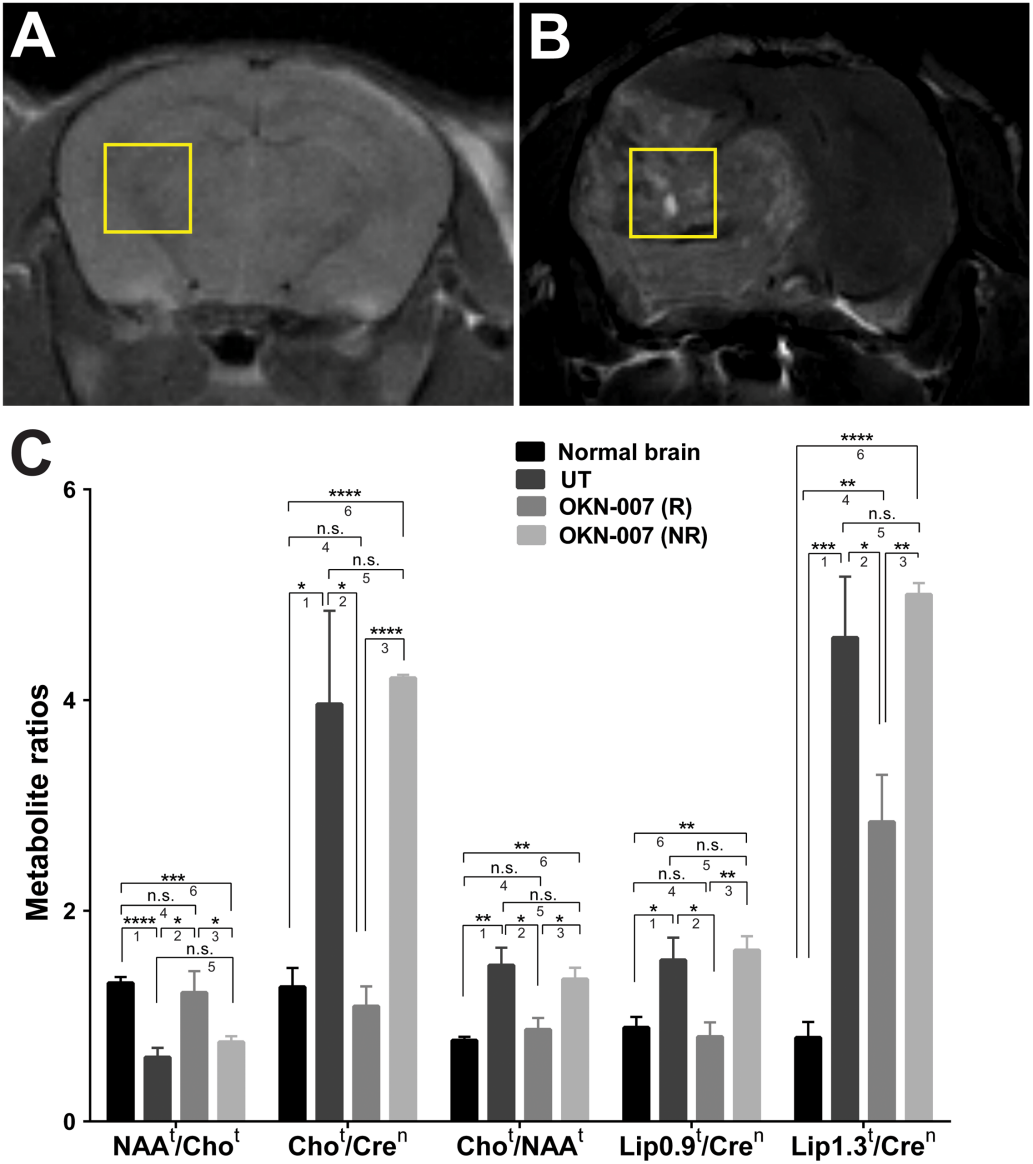
Brain metabolite ratios in normal and untreated vs. OKN-007 treated IC3752 pGBM tumor bearing mice. Representative locations of the 2 x 2x 2 mm3 voxels used to perform 1H-MRS in normal mouse brain (A) and IC3752 pGBM tumor bearing mice (B). (C) The untreated IC3752 pGBM mice demonstrated a significantly lower NAAt/Chot ratio and higher Chot/Cren (*p* = 0.0325), Chot/NAAt, Lip0.9t/Cren and Lip1.3t/Cren ratios when compared to the normal athymic mouse brains. The Lip1.3t/Cren, Lip0.9 t/Cren, Chot/NAAt, and Chot/Cren ratios were significantly lower in the OKN-007-(R) group when compared to untreated pGBM at the end phase of tumor progression. The OKN-007-(R) group showed a significantly higher NAAt/Chot ratio than the untreated pGBM. There was no significant difference between the Chot/Cren, NAAt/Chot, Chot/NAAt, and Lip0.9t/Cren ratios for the normal mouse brains compared to the OKN-007-(R) group. The OKN-007-(R) group showed a significantly higher Lip1.3t/Cren ratio than the normal mouse brain. There was no significant difference between the NAAt/Chot, Chot/Cren, Chot/NAAt, Lip0.9t/Cren and Lip1.3t/Cren ratios for the untreated compared to the OKN-007-(NR) groups. Asterisks indicate statistically significant difference (*p < 0.05; ***p<0.01; ***p<0.001; ****p<0.0001). Group comparisons: (1) Normal vs. UT; (2) UT vs. OKN-007 (R); (3) OKN-007 (R) vs OKN-007 (NR); (4) Normal vs. OKN-007 (R); (5) UT vs. OKN-007 (NR); and (6) Normal vs. OKN-007 (NR).

For all other investigations, OKN-007-(R) and OKN-007-(NR) treatment groups were compared to untreated tumor-bearing mice, as not all mice underwent ^1^H-MRS, DWI, perfusion imaging or IHC assessments. ^1^H-MRS values were obtained in normal athymic nude mice (n = 7), and untreated (n = 6) and OKN-007–treated (n = 8) mice bearing IC-3752GBM gliomas. Fig 4 shows the regions where the single 8mm^3^ voxel for ^1^H-MR spectroscopy was placed in the brains of normal mice (Fig 4A) and mice bearing IC-3752GBM gliomas (Fig 4B). Fig 4C shows that the untreated IC-3752GBM pGBM mice demonstrated significantly lower NAA^t^/Cho^t^ (*p* = 0.0001) and higher Cho^t^/Cre^n^ (*p* = 0.0325), Cho^t^/NAA^t^ (*p* = 0.0075), Lip0.9^t^/Cre^n^ (*p* = 0.0363) and Lip1.3^t^/Cre^n^ (*p* = 0.0009) ratios when compared to the normal athymic mouse brain. The Lip1.3^t^/Cre^n^ (*p* = 0.0395), Lip0.9^t^/Cre^n^ (*p* = 0.0257), Cho^t^/NAA^t^ (*p* = 0.0167), and Cho^t^/Cre^n^ (*p* = 0.0254) ratios were significantly lower in the OKN-007-(R) group than untreated pGBM at the end phase of tumor progression. The OKN-007-(R) group showed a significantly higher NAA^t^/Cho^t^ (*p* = 0.0492) ratio than the untreated pGBM. There was no significant difference between the Cho^t^/Cre^n^ (*p* = 0.498), NAA^t^/Cho^t^ (p = 0.6912), Cho^t^/NAA^t^ (*p* = 0.4231), and Lip0.9^t^/Cre^n^ (*p* = 0.6205) ratios of the normal mouse brain compared to the OKN-007-(R) group. The OKN-007-(R) group showed a significantly higher Lip1.3^t^/Cre^n^ (*p* = 0.0047) ratio than the normal mouse brain. There was no significant difference between the NAA^t^/Cho^t^ (*p* = 0.2071), Cho^t^/Cre^n^ (p = 0.7101), Cho^t^/NAA^t^ (*p* = 0.5362), Lip0.9^t^/Cre^n^ (*p* = 0.7277) and Lip1.3^t^/Cre^n^ (*p* = 0.5163) ratios of the untreated group compared to the OKN-007-(NR) group.

**Fig 5:**
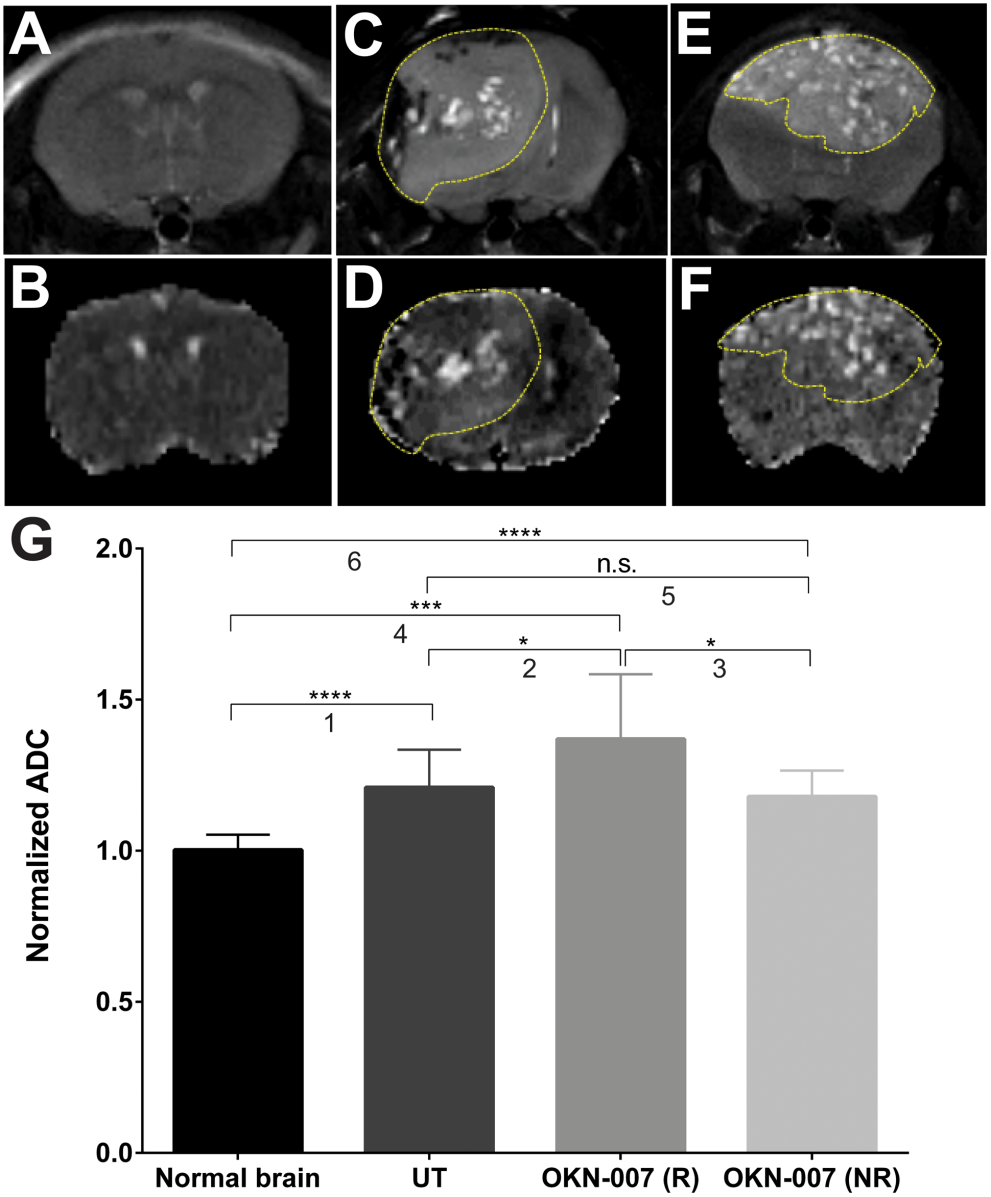
Effect of OKN-007 on diffusion measured as ADC values in IC3752 pGBM tumor bearing mice. T2-images (top) and diffusion maps (bottom) of normal mouse brain (A-B), untreated IC3752 pGBM (C-D), and OKN-007-R treated animal (E-F). (G) The untreated group (n = 11) had significantly higher normalized ADC values for the tumor region when compared to the normal mice brains (n = 6). The OKN-007–R treated group showed significantly higher normalized ADC values (n = 7) compared to the untreated group and normal mice brains. Values are represented as means ± SD. Asterisks indicate statistically significant difference (*p < 0.05; ***p<0.01; ***p<0.001; ****p<0.0001). Group comparisons: (1) Normal vs. UT; (2) UT vs. OKN-007 (R); (3) OKN-007 (R) vs. OKN-007 (NR); (4) Normal vs. OKN-007 (R); (5) UT vs. OKN-007 (NR); and (6) Normal vs. OKN-007 (NR).

In our study, with the use of DWI, the untreated group showed significantly higher (*p* = 0.0060) (1.161 ± 0.04538, n = 11) normalized ADC values in the tumor regions when compared to the normal mice brains (1.003 ± 0.009358, n = 6) (Fig 5A–5G). The OKN-007–R treated group had significantly higher normalized ADC values (1.296 ± 0.04141, n = 7) compared to the untreated group (p = 0.0432) or the normal mice brains (*p* = 0.0003). All OKN-007 treated animals were found to be responsive to the anticancer therapy based on the ADC ratios when compared to the untreated group (Fig 5G).

**Fig 6:**
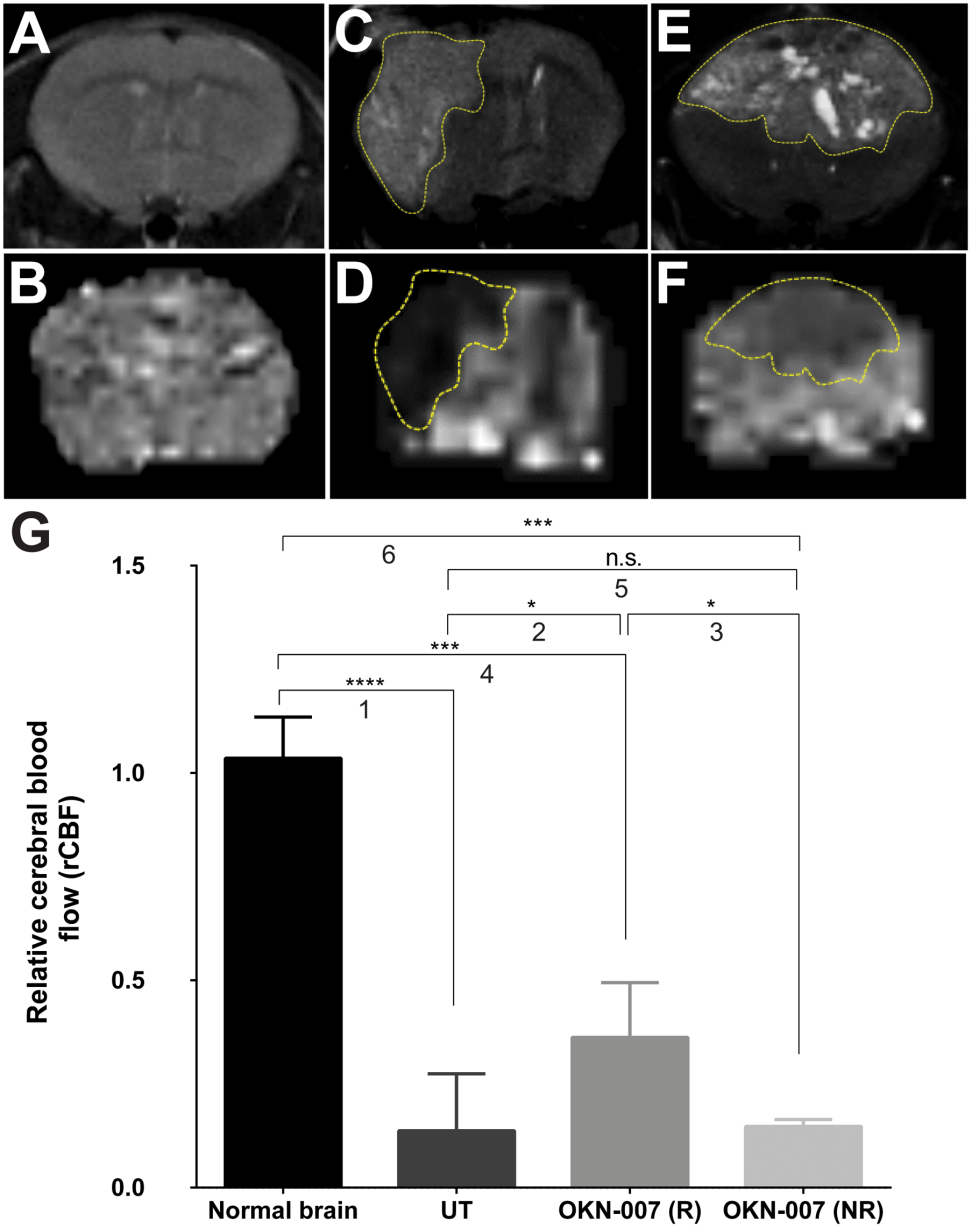
Effect of OKN-007 on perfusion rates measured as rCBF in IC3752 pGBM tumor bearing mice. T2-images (top) and perfusion maps (bottom) of normal mouse brain (A-B), untreated IC3752 pGBM (C-D), and OKN-007-R treated animal (E-F). (G) The untreated group (n = 9) showed significantly lower rCBF values for the tumor regions when compared to the normal mice brains (n = 9). The OKN-007–R treated group (n = 4) showed significantly higher rCBF values compared to the untreated group, and was significantly lower than normal brain. Values are represented as means ± SD. Asterisks indicate statistically significant difference (*p < 0.05; ***p<0.01; ***p<0.001; ****p<0.0001). n.s.: not statistically significant. Group comparisons: (1) Normal vs. UT; (2) UT vs. OKN-007 (R); (3) OKN-007 (R) vs. OKN-007 (NR); (4) Normal vs. OKN-007 (R); (5) UT vs. OKN-007 (NR); and (6) Normal vs. OKN-007 (NR).

When using perfusion imaging, the untreated group showed significantly lower (*p* < 0.0001) (0.1372 ± 0.04581, n = 9) rCBF values for the tumor regions when compared to the normal mice brains (1.036 ± 0.03305, n = 9) (Fig 6A–6G). The OKN-007–R treated group had significantly higher rCBF values (0.3624 ± 0.06599, n = 4) compared to the untreated group (*p* = 0.0307) or lower than the normal mice brains (*p* = 0.0004). There was no significant difference between rCBF values in the contralateral regions of OKN-007-R [104.8 ± 30.16 mL/(100g x min), n = 4] and OKN-007-NR [111.9 ± 34.15 mL/(100g x min), n = 2] or untreated [99.17 ± 19.50 mL/(100g x min), n = 7] animals (Fig 6G).

**Fig 7:**
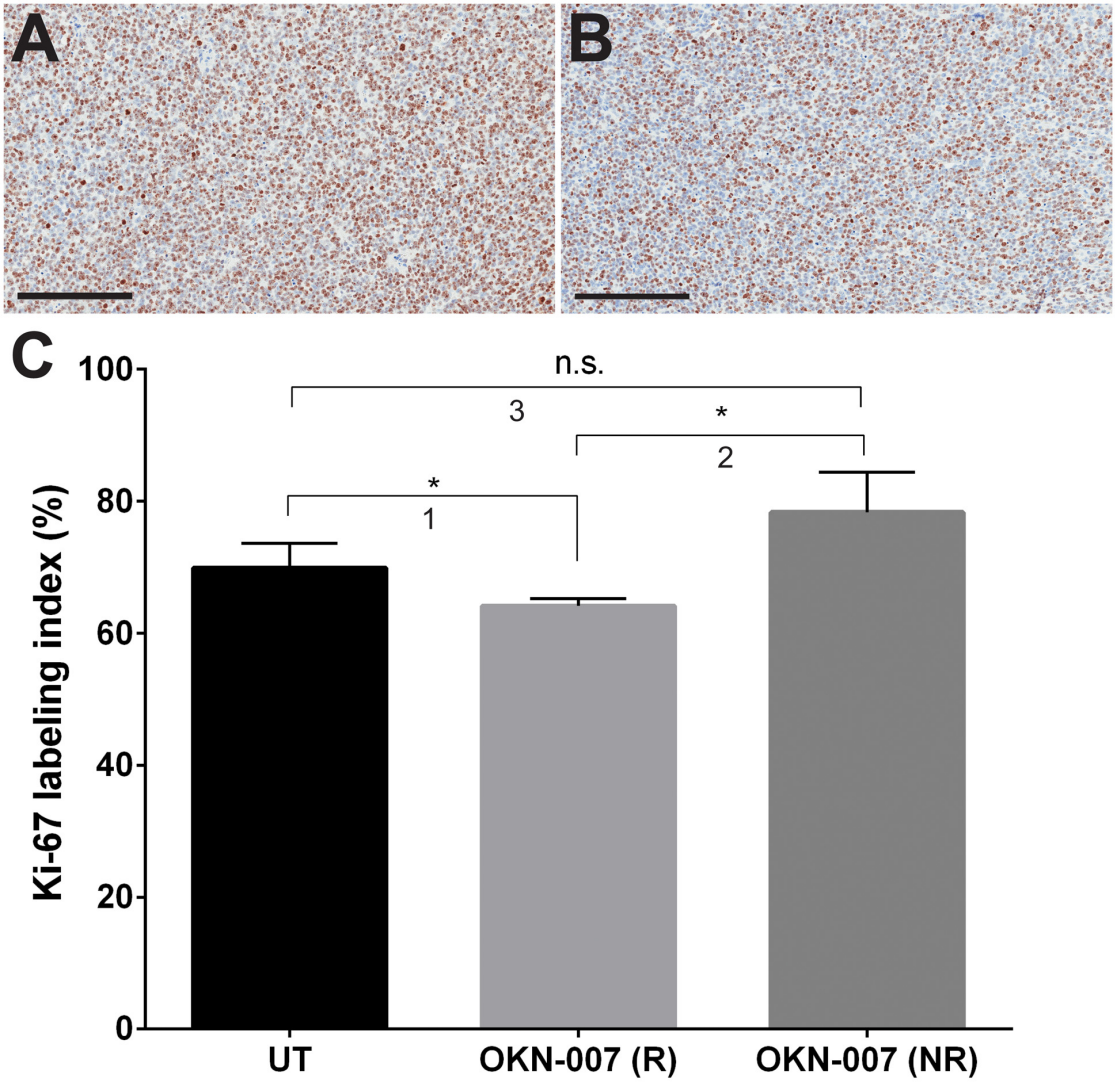
Effect of OKN-007 on cell proliferation in IC3752 pGBM tumor bearing mice. Immunohistochemistry images of Ki-67 levels in untreated (A) and OKN-007-treated (B) IC3752 pGBM tumors. (C) Ki-67 LI was significantly lower (*p* < 0.05) in the OKN-007–R treated group (n = 4) than in the untreated group (n = 6). There was no significant difference between the Ki-67 LI of the OKN-007–NR (n = 2) and the untreated groups. Values are represented as means ± SD. Asterisks indicate statistically significant difference (*p < 0.05). n.s.: not statistically significant. Scale bar = 200 μm. Group comparisons: (1) UT vs. OKN-007 (R); (2) OKN-007 (R) vs. OKN-007 (NR); and (3) UT vs. OKN-007 (NR).

Tumor cell proliferation was evaluated via immunoexpression of the anti-Ki-67 antibody. The Ki-67 LI was significantly lower (*p* = 0.0181) in the OKN-007–R treated group (64.15 ± 0.5322, n = 4) than in the untreated group (69.93 ± 1.524, n = 6) (Fig 7A–7C). There was no significant difference (*p* = 0.1822) between the Ki-67 LI of the OKN-007–NR (78.33 ± 4.287, n = 2) and untreated group.

**Fig 8:**
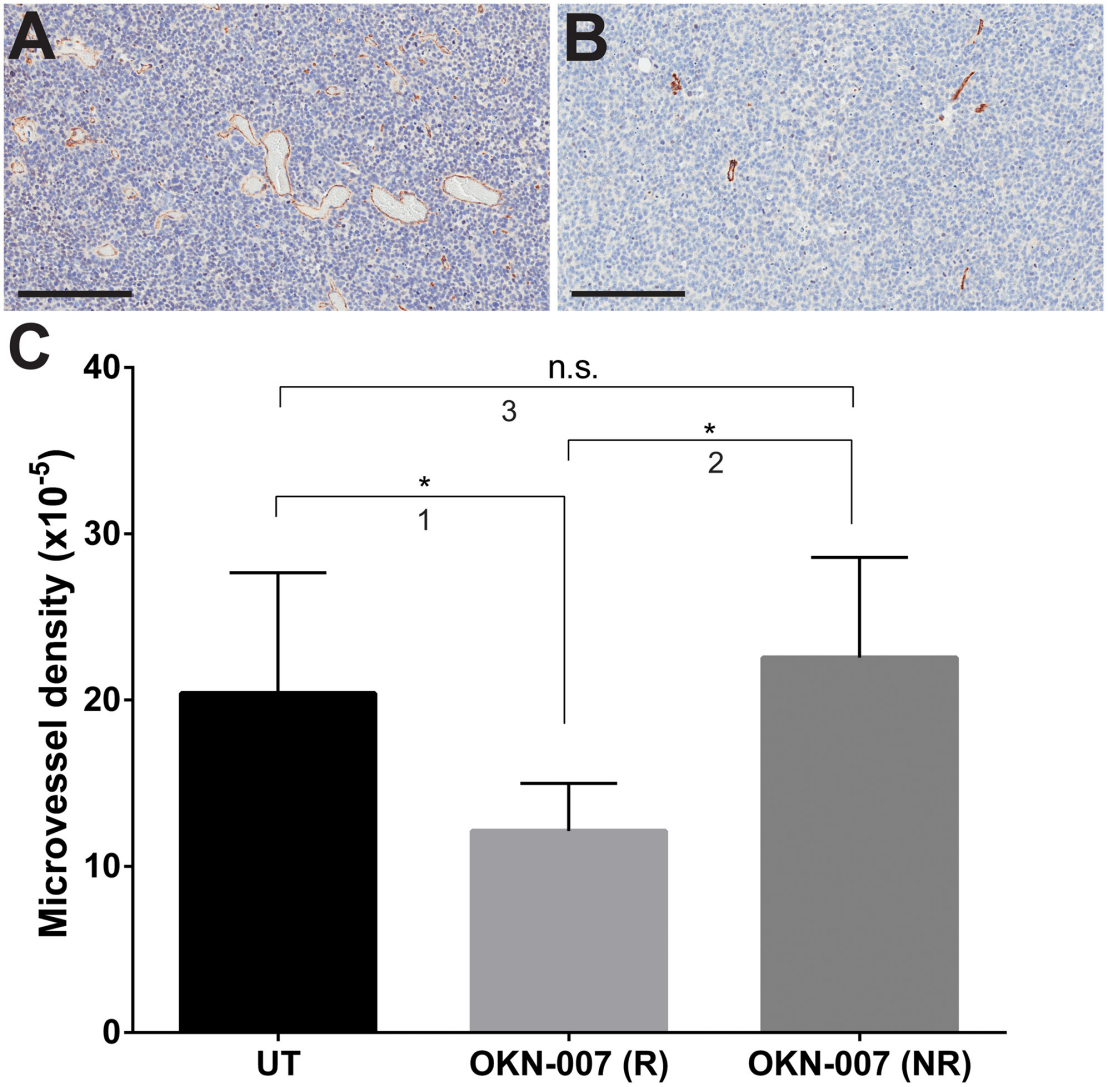
Effect of OKN-007 on microvessel density in IC3752 pGBM tumor bearing mice. Microvessel density (MVD) was measured via the immuno-expression of CD31. Immunohistochemistry images of CD31 levels in untreated (A) and OKN-007-treated (B) IC3752 pGBM tumors. (C) The MVD was significantly lower in the OKN-007–R treated group (n = 3) than in the untreated group (n = 7). There was no significant difference between the MVD for the OKN-007–NR (n = 4) and the untreated groups. The MVA was significantly lower in the OKN-007–R treated group (n = 3) than in the untreated group (n = 9). There was no significant difference between the MVA of the OKN-007–NR (n = 3) and the untreated groups. Values are represented as means ± SD. Asterisks indicate statistically significant difference (*p < 0.05). n.s.: not statistically significant. Scale bar = 200 μm. Group comparisons: (1) UT vs. OKN-007 (R); (2) OKN-007 (R) vs. OKN-007 (NR); and (3) UT vs. OKN-007 (NR).

Microvessel density (MVD) were measured via the immunoexpression of the anti-CD31 antibody. The MVD was significantly lower (*p* = 0.0358) in the OKN-007–R treated group (12.13 ± 1.648, n = 3) than in the untreated group (20.41 ± 2.737, n = 7) (Fig 8A–8C). There was no significant difference (*p* = 0.3092) between the MVD of the OKN-007–NR (22.55 ± 3.014, n = 4) and untreated group.

**Fig 9:**
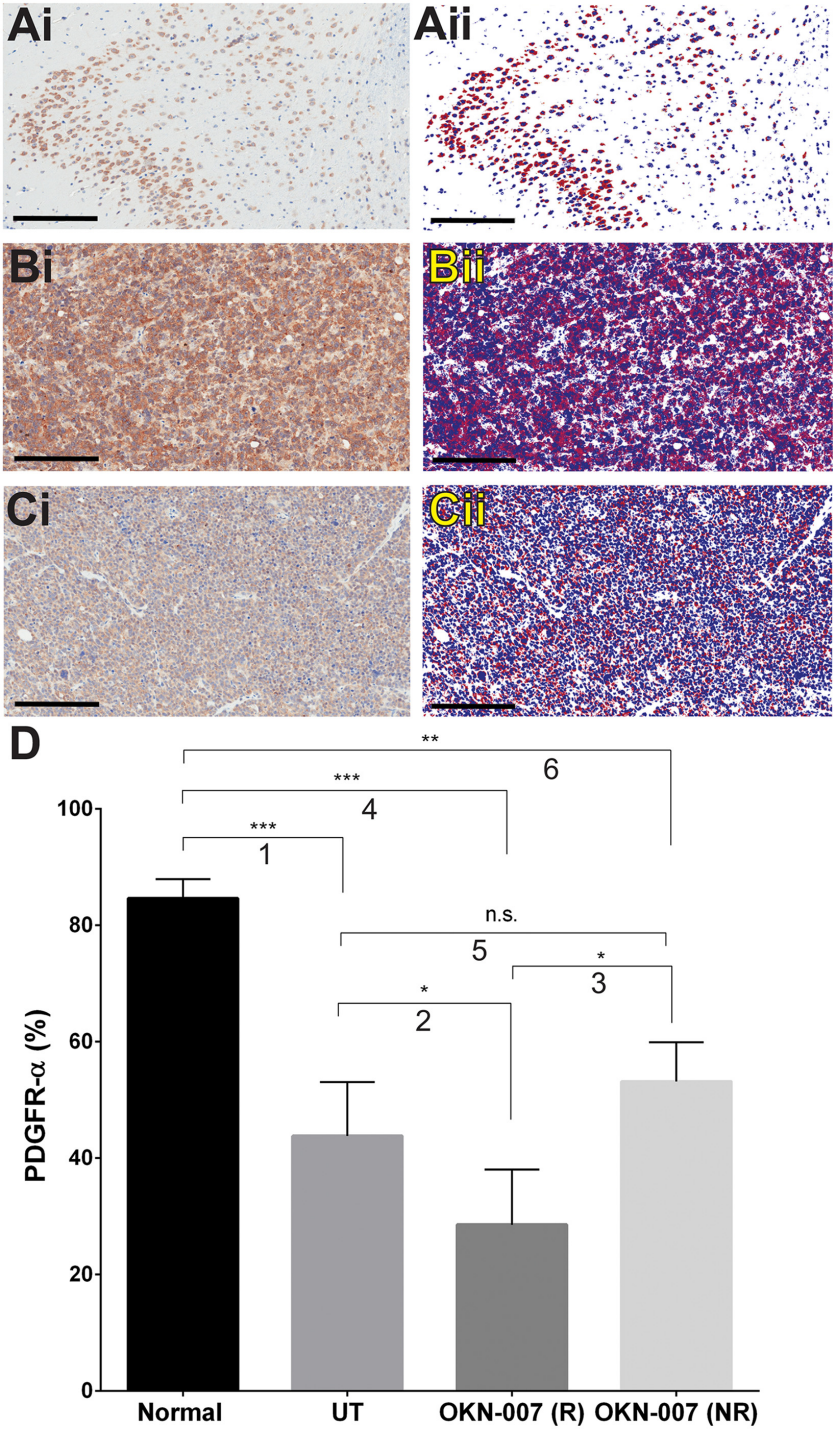
Effect of OKN-007 on the immunoexpression of PDGFR-α in IC3752 pGBM tumor bearing mice. (Ai-Ci) Immunohistochemistry expression of PDGFR-α in normal mouse brain, IC3752 pGBM UT, and OKN-007 treated animals, respectively. (Aii-Cii) Make up images for the immunoexpression of PDGFR-α in normal mouse brain, IC3752 pGBM UT, and OKN-007 treated animals, respectively. (D) OKN-007 significantly decreased the immunoexpression of PDGFR-α (%) in the treated responsive group (n = 4) when compared to the untreated animals (n = 6). There was no significant difference between the immunoexpression of PDGFR-α of the OKN-007–NR (n = 3) and the untreated groups. % PDGFR-α levels in high-expression areas within the normal brain tissue were significantly higher than those in UT or OKN-007-treated mice. Values are represented as means ± SD. Asterisks indicate statistically significant difference (*p < 0.05; **p<0.01; ***p<0.001). n.s.: not statistically significant. Red = positive, blue = negative, white = neutral, background. Scale bar = 200 μm. Group comparisons: (1) Normal vs. UT; (2) UT vs. OKN-007 (R); (3) OKN-007 (R) vs. OKN-007 (NR); (4) Normal vs. OKN-007 (R); (5) UT vs. OKN-007 (NR); and (6) Normal vs. OKN-007 (NR).

OKN-007 significantly decreased the immunoexpression of PDGFR-α in the treated responsive group (*p* = 0.0429) (28.58 ± 4.743, n = 4) when compared to the untreated animals (43.82 ± 3.779, n = 6). There was no significant difference (*p* = 0.1400) between the immunoexpression of PDGFR-α of the OKN-007–NR (53.17 ± 3.905, n = 3) and the untreated groups (Fig 9). Normal brain % PDGFR-α was found to be quite high in areas of tissue where ROIs were taken from elevated expression levels, and were significantly higher than the untreated or OKN-007-treated groups. The high % PDGFR-α levels in the normal brain are due to expression in both the nucleus and cytoplasm, whereas the majority of tumor cells had negative nuclei. Overall in Fig 9A it can be seen that levels of PDGFR-α are substantially lower than untreated tumors due to a 21% decrease in cell density which is 972.8 ± 85.8 mm^2^ (n = 5) for normal brain compared to 4620 ± 379 mm^2^ (n = 5) for untreated tumors.

**Fig 10:**
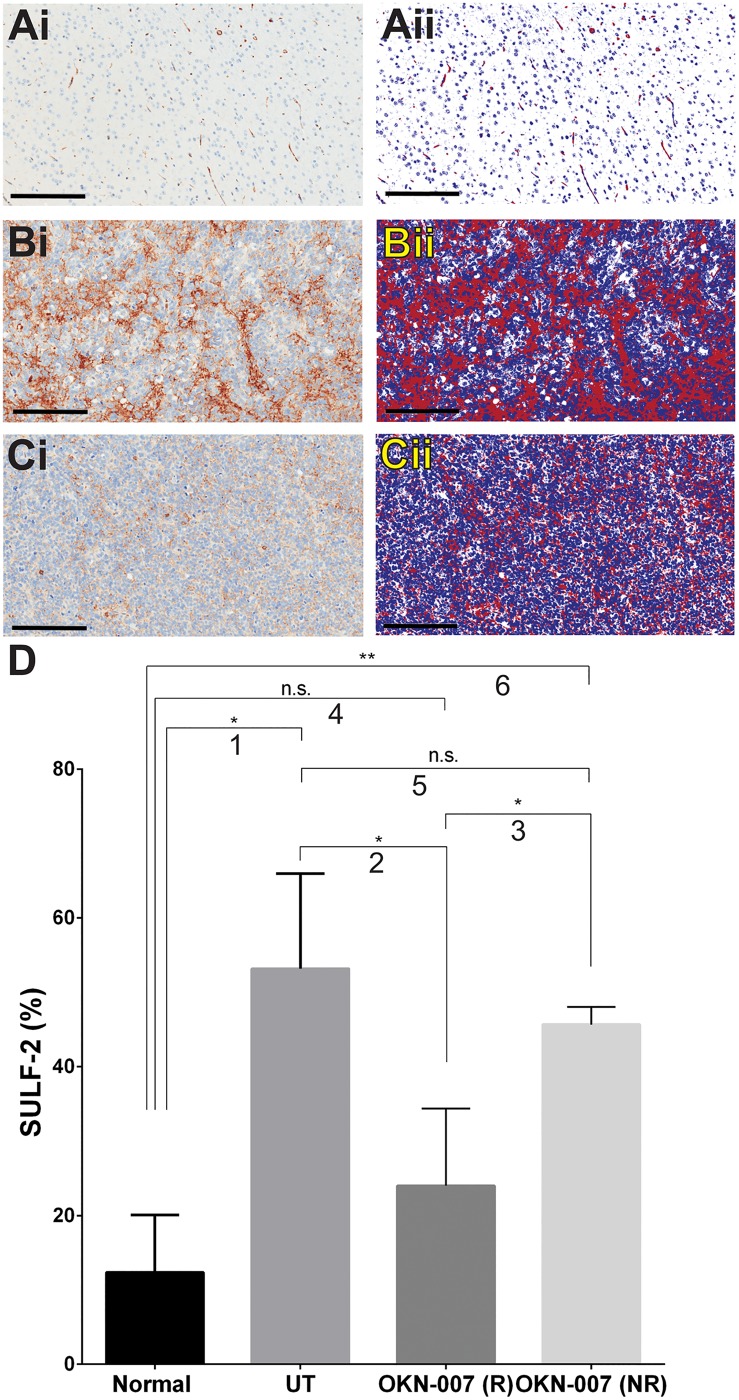
Effect of OKN-007 on the immunoexpression of SULF2 in IC3752 pGBM tumor bearing mice. (Ai-Ci) Immunohistochemistry expression of SULF2 in normal mouse brain, IC3752 pGBM UT, and OKN-007 treated animals, respectively. (Aii-Cii) Make up images for the immunoexpression of SULF2 in normal mouse brain, IC3752 pGBM UT, and OKN-007 treated animals, respectively. (D) OKN-007 significantly decreased the immunoexpression of SULF2 (%) in the treated responsive group (n = 4) when compared to the untreated animals (n = 3). There was no significant difference between the immunoexpression of SULF2 of the OKN-007–NR (n = 2) and the untreated groups. % SULF-2 levels in high-expression areas within the normal brain tissue were significantly lower than those in UT or OKN-007-treated mice. Values are represented as means ± SD. Asterisks indicate statistically significant difference (*p < 0.05; **p < 0.01). n.s.: not statistically significant. Red = positive, blue = negative, white = neutral, background. Scale bar = 200 μm. Group comparisons: (1) Normal vs. UT; (2) UT vs. OKN-007 (R); (3) OKN-007 (R) vs. OKN-007 (NR); (4) Normal vs. OKN-007 (R); (5) UT vs. OKN-007 (NR); and (6) Normal vs. OKN-007 (NR).

OKN-007 also significantly decreased the immunoexpression of SULF2 in the treated responsive group (*p* = 0.0339) (23.99% ± 5.187, n = 4) when compared to the untreated animals (53.17% ± 7.389, n = 3). There was no significant difference (*p* = 0.4180) between the immunoexpression of SULF2 of the OKN-007–NR (45.66% ± 1.686, n = 2) and the untreated groups (Fig 10). The levels of % SULF-2 in high-expression regions of normal brain were significantly less than that found in untreated or OKN-007-treated tumors. The expression of SULF-2 was significantly decreased in the normal brain because it was expressed predominantly in the endothelial cells. In this case, the number of negative pixels was higher, and consequently the positivity (Positivity = Positive / Positive + Negative) was decreased.

**Fig 11:**
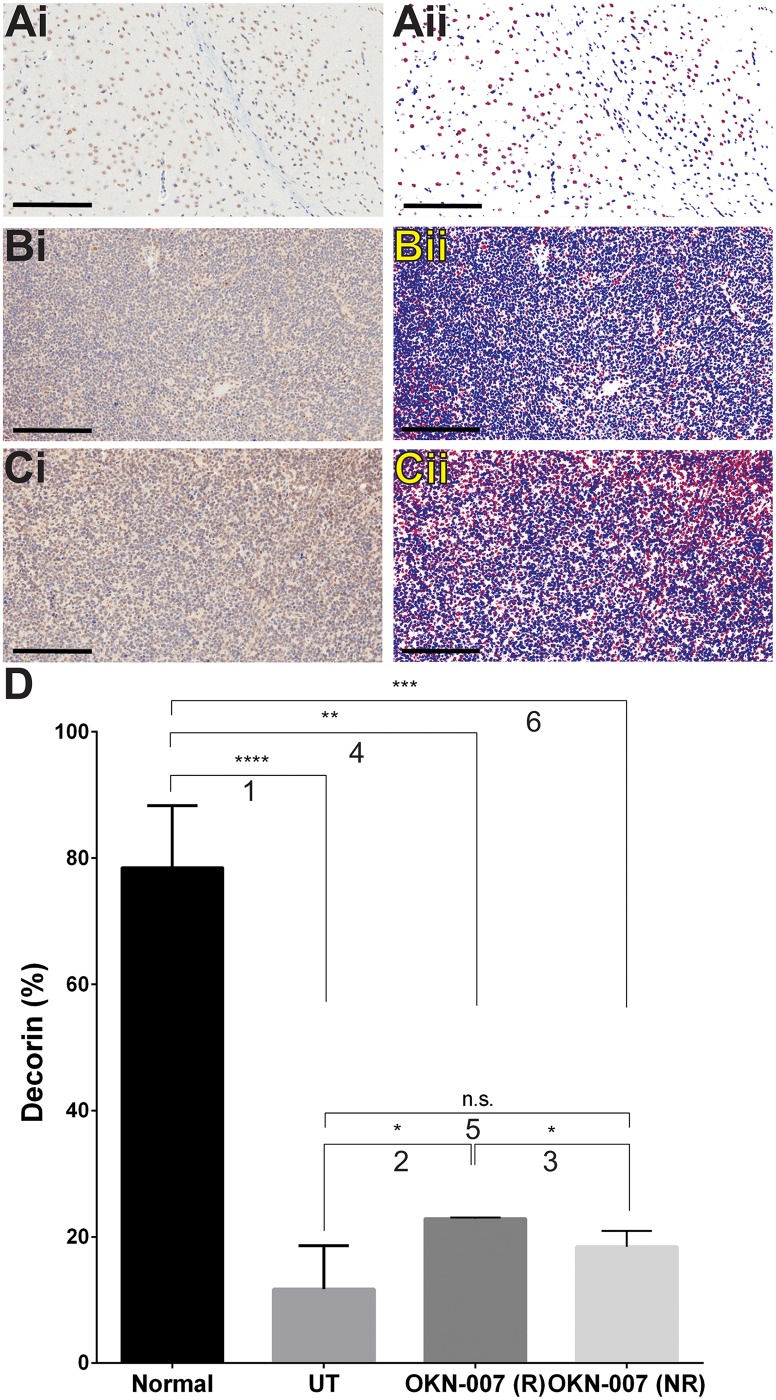
Effect of OKN-007 on the immunoexpression of decorin in IC3752 pGBM tumor bearing mice. (Ai-Ci) Immunohistochemistry expression of decorin in normal mouse brain, IC3752 pGBM UT, and OKN-007 treated animals, respectively. (Aii-Cii) Make up images for the immunoexpression of decorin in normal mouse brain, IC3752 pGBM UT, and OKN-007 treated animals, respectively. (D) The immunoexpression of decorin was significantly higher (*p* = 0.0478) in the OKN-007–R treated group (22.86 ± 0.1479, n = 2) compared to the untreated animals (11.73 ± 3.438, n = 4). There was no significant difference (*p* = 0.0167) between the immunoexpression of decorin for the OKN-007–NR (18.44 ± 1.128, n = 5) and the untreated groups. % Decorin levels in high-expression areas within the normal brain tissue were significantly higher than those in UT or OKN-007-treated mice. Values are represented as means ± SD. Asterisks indicate statistically significant difference (*p < 0.05; **p<0.01; ***p<0.001; ****p<0.0001). n.s.: not statistically significant. Red = positive, blue = negative, white = neutral. Scale bar = 200 μm. Group comparisons: (1) Normal vs. UT; (2) UT vs. OKN-007 (R); (3) OKN-007 (R) vs. OKN-007 (NR); (4) Normal vs. OKN-007 (R); (5) UT vs. OKN-007 (NR); and (6) Normal vs. OKN-007 (NR).

The immunoexpression of decorin was significantly higher (p = 0.0478) in the OKN-007–R treated group (22.86 ± 0.1479, n = 2) compared to the untreated animals (11.73 ± 3.438, n = 4). There was no significant difference (*p* = 0.0167) between the immunoexpression of decorin for the OKN-007–NR (18.44 ± 1.128, n = 5) and the untreated groups (Fig 11). The levels of % decorin in high-expression regions of normal brain were significantly higher than that found in untreated or OKN-007-treated tumors. The high % decorin levels in the normal brain are due to expression in both the nucleus and cytoplasm, similar to PDGFR-α, and conversely the majority of tumor cells had negative nuclei. Also taking into consideration the 21% decrease in cell density in normal brain compared to untreated tumors, the overall levels of decorin would be less overall when considering all areas of tissue.

## Discussion

Animal modeling for primary brain tumors has undergone constant development over the last 60 years, and significant improvements have been made recently with the establishment of highly invasive glioblastoma models through the direct implantation of patient tumors [9,44]. However, animal models for pediatric HGGs are still an underexploited tool to make rapid advances in human cancer prevention, diagnosis, and therapy.

This is the first description of the macroscopy, histology, immunohistochemistry and MRI characterizations of the novel orthotopic xenograft pediatric glioblastoma (pGBM) model IC-3752GBM [43], and assessing the ability of OKN-007 to inhibit IC-3752GBM tumor cell growth. Previous reports in GBM preclinical models are limited to *in vitro* ^1^H-MRS in other pGBM cell lines [12].

The anti-cancer response to OKN-007 in adult glioma models was previously reported by our group based on morphological (T_2_-weighted) imaging, bioluminescence imaging, and immunohistochemistry assessments, in addition to survival data [11,15,16]. The cytotoxic mechanism of OKN-007 seems to be associated with induced apoptosis in rat F98 and human U87 xenografts [16]. OKN-007 was found to significantly decrease tumor volumes (*p* < 0.05), as measured by MRI, and increase survival (*p* < 0.001) in F98 glioma-bearing rats [23]. Assessment of the treatment response using other MR parameters, such as ^1^H-MRS, DWI, and perfusion rates, was also performed by our group in C6 rat gliomas [10,15], but the use of a multi-parametric approach has not been previously reported for any pediatric glioblastoma model.

From gross examination of the IC-3752GBM pGBM (Fig 1A–1B), the tumors were characterized as a yellowish gray, poorly demarcated mass, with multifocal areas of necrosis. Histological examination (Fig 1C–1E) revealed poorly demarcated, unencapsulated infiltrating neoplasms with marked cell atypia, a high mitotic index, and multifocal areas of necrosis and hemorrhaging, which is comparable to features for GBM in pediatric patients [2]. We previously demonstrated that serial passage (up to six times) of patient-derived xenografts of IC-3752GBM pGBM replicated the histological features and genetic changes (e.g. loss of heterozygosity) associated with the original patient tumor [44].

The survival time of IC-3752GBM pGBM mice was significantly increased in all treated mice (p<0.05) and the responsive group (p<0.001) after OKN-007 therapy, as we also have previously reported in adult glioma models [15,16]. The total OKN-007 group had a median survival of 25.5 days, whereas the OKN-007-R had a mean survival time of 33 days, which were both longer than the median survival of 19.5 days for the untreated group (Fig 2). In our study, OKN-007 was able to increase the survival time by 6 days compared to the untreated animals. Considering that one mouse month is estimated to be ∼2.6 human years, based on a 4-year maximum life span [47,48], the increased survival time in human patients could be estimated to be ~6–7 months.

From contrast-enhanced *in vivo* morphological MR imaging (Fig 3B), IC-3752GBM pGBM tumors were characterized as mildly to markedly hyperintense, with poorly defined margins, and infiltrative tumors, sharing similar MRI characteristics with human glioblastomas [49]. Morphological MR images were also used to determine the volumes of individual tumors, starting on day 25 after the cell implantation, and continuing throughout the entire experiment. For the entire OKN-007-treatment group, there was a significant decrease in tumor volumes (p<0.05), compared to untreated mice. Additionally, the therapeutic responses to OKN-007 were evaluated as either responsive (OKN-007-R) or unresponsive (OKN-007-NR). One cohort of mice were found to respond to OKN-007 treatment [OKN-007-(R)], based on survival data, and demonstrated significantly smaller tumors (*p* <0.001) when compared with untreated animals (Fig 3E). However, there were also non-responsive mice [OKN-007-(NR)] that seemed to be not affected to the OKN-007 therapy. For the responsive mice, our results were similar to prior adult glioma studies of our group, which showed that OKN-007 had a dramatic effect on regressing tumor formation in rat F98 [16] and C6 [11,15], and human U87 xenograft [16] glioma models.

A possible explanation for the varied treatment response may be due to the heterogeneous nature of the cell population that is being passaged from mice to mice, unlike a homogeneous cell culture population like what was used in our adult glioma studies. From this study it is clear that not all of the cells respond appropriately to OKN-007 treatment. Possibly there is a resistance that develops in some of the cells which overtake the growth of the responsive cells in the OKN-007-NR group, whereas in the OKN-007-R group the responsive cells predominate. Further cell sorting studies could be done to isolate these two cell populations, however distinctive markers that differentiates these two cell types would need to be sought.

We also assessed the OKN-007 treatment response by using other MR parameters, such as ^1^H-MRS to measure tumor metabolites, DWI to assess structural alterations, and perfusion rates to evaluate microvascularity alterations. ^1^H-MRS is a non-invasive technique that measures the concentration of a variety of biomolecules from a volume of interest [50].

MRS findings have been shown to be closely related to the histological features of glioma cells and can be used for evaluating tumor differentiation, grading, follow-up and radiotherapy planning [51–53]. MRS is also a valuable tool for identifying early changes in glioma metabolism and the extent of glioma infiltration [54,55]. The technique is widely available clinically and easily appended to a standard MRI examination, which is routinely performed at the time of diagnosis on children with brain tumors [56]. The principal metabolites that can be assessed by ^1^H-MRS in brain tumors include choline (Cho, 3.2 ppm), creatine (Cre, 3.0 ppm), N-acetylaspartate (NAA, 2.0 ppm), and lipids (Lip; 0.9–1.3 ppm) [55]. Choline is a constituent molecule associated with the phospholipid metabolism of cell membranes and reflects membrane turnover [57]. Its concentration is slightly greater in white matter than in gray matter [57]. Increased Cho indicates greater membrane synthesis and cell proliferation [57]. Its concentration is markedly increased in cases of brain neoplasms [57]. Phosphocholine and glycerophosphocholine also contribute to the representation of the Cho peak in ^1^H-MRS [57]. Creatine is a marker of aerobic energy metabolism in brain cells, and is present in larger concentrations in the gray matter than in the white [57]. Creatine and phosphocreatine, both of which contribute to the Cre pool, are found in both neurons and glial cells [57]. Cre and phosphocreatine remain relatively stable *in vivo*, and therefore their peak is often used as an internal concentration standard [58]. However, recent studies have suggested that the Cre levels may be altered in human gliomas [59]. N-acetyl aspartate, a free amino acid, is almost exclusively located in neurons and axons, and is the most prominent signal observed in ^1^H-MRS for the CNS. The NAA peak in ^1^H-MRS is decreased whenever there is neuron loss, such as in gliomas, ischemia and degenerative diseases [57]. A strong association between ^1^H-MRS detectable lipids and tumor grade has been reported in adult gliomas [60] and childhood brain tumors [61] suggesting that high intracellular lipid levels are a non-invasive marker of brain tumor malignancy. Studies on cultured cells, *ex-vivo* tumor tissue and animal models have shown that an increase in MRS detectable lipids is associated with cell stress [62], apoptosis [63] and hypoxia as a result of compromised vascularity [64]. MRS detectable lipids are therefore associated with several factors known to be present in aggressive tumors that have a poor prognosis.

In our study, we measured the peak areas of Cho, Cre, NAA, Lip0.9, and Lip1.3 in the normal brain of athymic nude mice and untreated and OKN-007 treated IC-3752GBM pGBM bearing mice. Those values were used to determine the following ratios: tumor NAA to tumor Cho (NAA^t^/Cho^t^), tumor Cho to contralateral Cre (Cho^t^/Cre^n^), tumor Cho to tumor NAA (Cho^t^/NAA^t^), tumor Lip0.9 to contralateral Cre (Lip0.9^t^/Cre^n^), and tumor Lip1.3 to contralateral Cre (Lip1.3^t^/Cre^n^). The untreated IC-3752GBM pGBM mice demonstrated significantly lower NAA^t^/Cho^t^ (*p* < 0.001) and higher Cho^t^/Cre^n^ (*p* < 0.05), Cho^t^/NAA^t^ (*p* < 0.01), Lip0.9^t^/Cre^n^ (*p* < 0.05) and Lip1.3^t^/Cre^n^ (*p* < 0.001) ratios when compared to the normal athymic mouse brain (Fig 4C), which corroborates with metabolite profiles observed in human GBM. Typical ^1^H-MRS findings for cerebral gliomas includes reduction of NAA, variable levels of Cr, and an elevation in Cho and lipids, in proportion to glioma grade [65].

The role of mobile lipids in cancer has been the subject of many excellent reviews and articles [10,11, 64,66–70]. In brain tumors, the elevation of lipid levels usually correlates with necrosis [66,67,71], and are considered as important biomarkers for diagnosis and monitoring the effects of treatment response [66]. A number of studies have also demonstrated that an increase in mobile lipids is associated with the onset of drug-induced apoptosis [62,66]. OKN-007 significantly reduced the lipid peaks at 1.3ppm (*p* < 0.05) and 0.9 ppm (*p* < 0.05) in treated animals compared to the untreated group, which could be related to a decrease in tumor necrosis by OKN-007 in treated animals. This anticancer property of OKN-007 may be very beneficial for GBM patients, since necrosis is caused by tumor hypoxia as a result of increased cell proliferation and mitotic activity, as well as insufficient tissue perfusion [72], and it is present in over 85% of cases [73,74].

The IC-3752GBM pGBM bearing mice treated with OKN-007 showed a significantly lower Cho^t^/NAA^t^ ratio (*p* < 0.05) than the untreated group. The effect of OKN-007 on the Cho/NAA ratio in the IC-3752GBM pGBM model is remarkable, since it has been reported that the Cho/NAA ratio provides a sensitive method for detecting differences in tumor growth [75]. Furthermore, baseline Cho/NAA ratios and the percent change in Cho/NAA over time have also been associated with poor prognosis in childhood brain tumors [76–78]. Lower Cho/NAA ratios seem to be predictive of the improved outcome in children with recurrent primary brain tumors, and should be evaluated as a prognostic indicator in newly diagnosed childhood brain tumors [76].

The Cho^t^/Cre^n^ ratios also significantly decreased (*p* < 0.05) after OKN-007 treatment in responsive animals compared to untreated pGBM at the end phase of tumor progression (Fig 4C), indicating that the drug was also capable of decreasing tumor cell proliferation in ~50% of animals in this pediatric glioma model. The ^1^H-MRS results were also confirmed with IHC that showed significantly lower (*p* < 0.05) immunoexpression of Ki-67 (cell proliferation marker) in the OKN-007–R treated group compared to untreated animals (Fig 7C).

Diffusion-weighted imaging (DWI) represents another promising imaging tool for tissue characterization, prediction, and the evaluation of therapeutic response in oncology [79]. DWI is routinely used to measure microscopic, random translational motion of water molecules within tissues. Any alterations in the tissue structure that disrupt the barriers of water diffusion, such as the breakdown of cell membranes or damage to fibers, would lead to changes in the diffusion properties within these regions. These diffusion characteristics can be quantified by estimating the ADC (Apparent Diffusion Coefficient). In tumors, ADC values can differentiate the cellular mechanisms involved in tumor development as well as responses to treatment such as cell proliferation, apoptosis, and/or necrosis [80]. Both intra- and extracellular spaces and their exchange contribute to the measured ADC [81]. DWI has also a tremendous potential for monitoring early changes in tumor cellularity that are thought to be reflective of treatment response [81]. As cellular density increases, the added tortuosity to extracellular mobility paths reduces water mobility and, consequently, the ADC value [82]. Processes that degrade cellular integrity, such as necrosis caused by therapy or tumor growth, are thought to increase the ADC of tissue [82]. Successful treatment of a tumor with a cytotoxic agent will result in significant damage to the tumor cells in the form of a loss of cell membrane integrity with a subsequent reduction in tumor cell density. This has a net effect of increasing the fractional volume of the interstitial space because of cell loss, resulting in an increase in the mobility (diffusion) of water within the damaged tumor tissue [81].

In our study, the untreated group showed significantly higher (*p* < 0.01) normalized ADC values for the tumor region when compared to the normal mice brain (Fig 5G). The OKN-007–R treated group showed significantly higher normalized ADC values than the untreated group (*p* < 0.05) and normal mice brains (*p* < 0.001). Interestingly, all OKN-007 treated animals were responsive to the therapy based on the ADC ratios when compared to the untreated group. The observed increase in water ADC following therapy is directly related to the number of tumor cells killed and is thought to be due to the liberation of water into the extracellular space [82].

Arterial spin labeling (ASL) is a non-invasive magnetic resonance technique for tissue perfusion quantification without the application of contrast media. Perfusion is defined as the amount of blood delivered to capillary beds in a given tissue per unit time. Delivery of blood to a capillary bed is a critical indicator of tissue viability and function, and the analysis of perfusion [83], which has markedly contributed to the understanding of physiological and pathological processes in the human body [84]. Furthermore, the ASL technique has been useful for monitoring the effects of agents designed to modulate tumor blood flow and oxygenation (e.g., carbogen gas) [85], and for evaluating and guiding the use of anti-angiogenic agents [42].

Antiangiogenic therapy might affect tumor vessels in 3 different ways: no effect at all; excessive destruction of blood vessels and a reduction in perfusion leading to increased hypoxia, necrosis, and/or invasion; or after pruning of some abnormal vessels the overall structure of the remaining tumor vessels may resemble normal vessels, which potentially results in an increase in absolute blood perfusion [86]. In this study, we measured the changes in normalized relative cerebral blood perfusion (rCBF) in normal athymic mouse brains, and in untreated and OKN-007 treated IC-3752GBM pGBM mice, using the ASL perfusion MRI technique. The untreated group showed significantly lower (*p* < 0.0001) rCBF values for the tumor region when compared to the normal mice brain (Fig 6G). The OKN-007–R treated group showed significantly higher rCBF values compared to the untreated group (*p* < 0.05) and significantly lower than normal mice brains (*p* < 0.001). Interestingly, the drug did not seem to affect the normal perfusion of the brain, as no differences were found between values in the contralateral regions of OKN-007-R and OKN-007-NR and untreated animals (Fig 6G), being consistent with the findings from previous research studies in OKN-007-treated C6 gliomas [22]. Our results also corroborate with the literature that indicates that glioblastomas have lower blood perfusion rates than that of the surrounding normal brain because of their inefficient, irregular vessels that are leaky and dilated with a haphazard pattern of interconnection [86].

The positive effect of OKN-007 on perfusion and diffusion properties in IC-3752GBM pGBM bearing mice indicates a promising influence of the drug on tumor microvasculature, which was also shown previously in a rat adult glioma model by our group [15]. In our study, we were also able to demonstrate that OKN-007 acts as an antiangiogenic agent in the IC-3752GBM pGBM model. To support this notion, microvessel density (MVD) was significantly lower (*p* < 0.05) in the OKN-007–R treated group than in the untreated group (Fig 8C). The normalization of tumor vasculature is a necessary step for cancer therapies [87]. This effect may be mediated by the anti-inflammatory properties of OKN-007. Indeed, alterations to inflammatory pathways can modify angiogenesis [88], and inflammatory mediators are known to modulate blood—brain barrier permeability [89]. Therefore it could be expected from the anti-inflammatory properties of nitrone compounds, that administration of OKN-007 positively altered the integrity and function of the microvascular bed at the tumor site, consequently increasing the ADC ratios and rCBF values in responsive treated animals.

We also assessed the effect of OKN-007 regarding the immunoexpression of tumor signaling molecules such as PDGFR*α*, SULF2, and decorin in the IC-3752GBM pGBM model. Although childhood and adult HGGs share related histopathological characteristics, recent studies have shown substantial differences in the molecular features underlying pediatric and adult HGGs. The epidermal growth factor receptor is the predominant receptor tyrosine kinase targeted by both amplification and mutation in adult glioblastomas [90]. In contrast, PDGFR*α* is the most frequent target of focal amplification in pediatric HGGs arising within and outside the brainstem [20–23], and somatic mutations of PDGFR*α* have been recently reported in pediatric HGGs [22–24]. Another study revealed that the high expression of phosphorylated PDGFR*α* has a significant association with malignant histology in pediatric gliomas [91]. The expression of PDGFR*α* has also been demonstrated in medulloblastomas and primitive neuroectodermal tumors, and an increase in PDGFR*α* gene copy numbers seems to be associated with poor survival in these tumors [92]. The high levels of % PDGFR-α observed in specific high-intensity ROIs may occur because the mice are 2–3 month-old mice which are still adolescents, and it is known that normal brain tissue may have some areas of high PDGFR-α during development [93].

SULF2, a heparan sulfate endosulfatase, has been associated to different types of cancers including lung [94], breast [95], liver [96], gastric [97], and brain tumors [98]. In brain cancer, SULF2 has been 7directly implicated in driving tumorigenesis in murine and human malignant gliomas [99]. The SULF2 protein is highly expressed in primary human GBM, and SULF2 levels are inversely related to heparan sulfate proteoglycan (HSPG) 6O-sulfation in a murine model for GBM [99]. SULF2 gene expression is also significantly positively correlated with PDGFR*α* expression [25]. Furthermore, the ablation of SULF2 decreases tumor growth, prolongs host survival and decreases the activity of PDGFR*α*, as well as related downstream signaling pathways in human and mouse malignant gliomas [99].

Decorin is a small proteoglycan found in the extracellular matrix of a variety of tissues and cell types [25,100]. It interacts with a diversity of proteins that are involved in matrix assembly [101] and in the regulation of fundamental biological functions such as cell attachment [102], migration [103], and proliferation [104]. Decorin inhibits cell proliferation in several tumor cell types, including ovarian [101], liver [26], and brain tumor cells [105]. Similarly to SULF2, decorin is also associated to the PDGFRα pathway [26]. Interestingly, decorin does not colocalize with PDGFRα, but binds directly to its natural ligand PDGF, directly blocking the activity of PDGFRα [104]. Furthermore, it is known that decorin antagonizes the angiogenic network [106] due to the inhibition of the production of VEGF (vascular endothelial growth factor) by tumor cells and can directly blocking of VEGFR2 (vascular endothelial growth factor receptor-2) at the same time [107].

**Fig 12:**
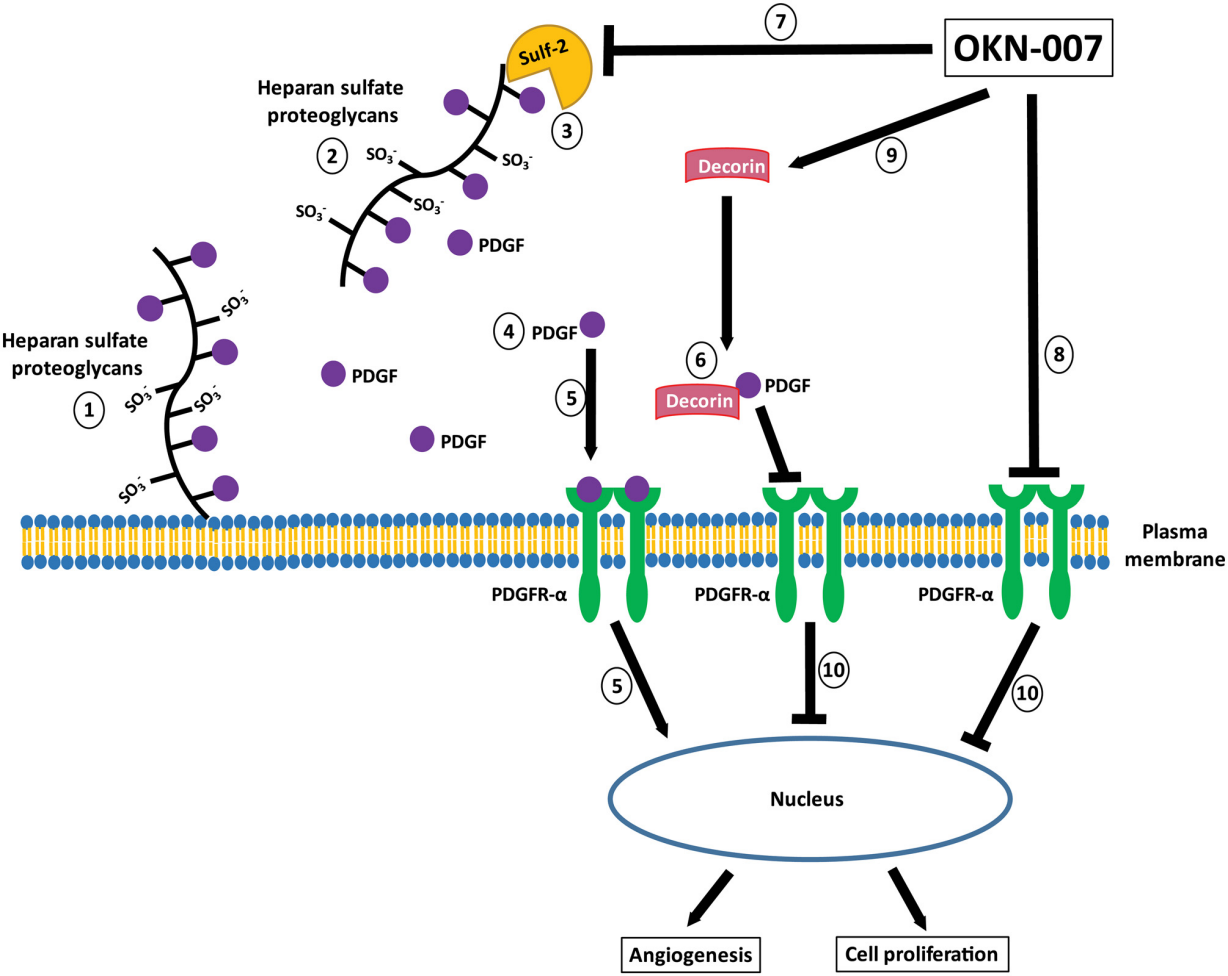
Schematic representation of possible mechanism of action of OKN-007 in IC3752 pGBM tumor bearing mice. Heparan sulfate proteoglycans (HSPG) consist of a protein core and heparan sulfate (HS) chains consisting of linear carbohydrate chains of repeating disaccharide units. Dependent on the HSPG core protein, HSPGs are found at the cell surface (1), in the extracellular matrix (2), or in secretory vesicles [98]. Essential for their function in cell signaling, the HS chains undergo extensive post-translational modifications, including sulfation on the 6-O- position of glucosamine [19]. By removing 6-O-sulfates on HS chains and mobilizing protein ligands from HSPG sequestration in the extracellular environment (3), the SULF-2 can increase the solubility of a variety of growth factors (e.g. PDGF) (4) thus increasing the activation of PDGFR-α and downstream signaling pathways in tumor cells (5) [98]. Similarly to SULF2, decorin is also associated to the PDGFRα pathway. Interestingly, decorin does not co-localize with PDGFRα, but binds directly to its natural ligand PDGF, directly blocking the activity of PDGFR*α* (6) [104]. Our current data showed that OKN-007 was able to decrease the immunoexpression of SULF2 (7) and PDGFR*α* (8), and increase the expression of decorin (9) in IC3752 pGBM tumor bearing mice, which may be also associated to the inhibition of tumor angiogenesis and cell proliferation (10) also observed in OKN-007 treated animals. SO3-: sulfate, PDGF: Platelet-derived growth factor. The schematic is a modification of combined schemes obtained from Oncotarget, 3(5): 568–575, Phillips JJ (2012) [98] (With permission from Impact Journals), and from FEBS Journal, 280(10):2150–2164, Baghy K et al., (2013) [104] (With permission from John Wiley and Sons).

Our current data showed that OKN-007 was able to significantly decrease the immunoexpression of SULF2 (*p* < 0.05) in responsive mice compared to untreated animals, which has also been recently demonstrated in hepatocellular cancer cells [19]. Furthermore, OKN-007 was able to significantly decrease the immunoexpression of PDGFR*α* (*p* < 0.05) and significantly increase the expression of decorin (*p* < 0.05) in responsive IC-3752GBM pGBM tumor bearing mice, compared to untreated animals, which may be also associated to the inhibition of tumor angiogenesis and cell proliferation which was also observed in the OKN-007-R treated animals from this study. Fig 12 depicts the possible mechanism-of-action of OKN-007 in pGBM.

## Conclusions

The data taken as a whole indicates that OKN-007 may possibly be an effective anti-cancer agent for some patients with pHGGs by inhibiting cell proliferation and angiogenesis, potentially via the PDGFR*α* pathway, and could be considered as an additional therapy for pediatric brain tumor patients. Currently, OKN-007 is an investigational drug in phase Ib/IIa clinical trials for recurrent adult GBM patients. Our future plan is to conduct clinical trials in pGBM patients using OKN-007 as an adjunct therapy to possibly help inhibit tumor growth associated with this devastating disease in children.
